# Kinesin-1 coordinates cross-talk between microtubule and actin cytoskeletons during dendritic cell migration

**DOI:** 10.1126/sciadv.adx7672

**Published:** 2025-10-17

**Authors:** Pierre Duquesne, Céline Aoun, Mathieu Kurowska, Brieuc P. Perot, Kerui Zhang, Mounia Debili, Mirjana Weimershaus, François-Xavier Mauvais, Nicolas Cagnard, Nicolas Goudin, Bernardita Medel, Juan Eduardo Montero-Hermández, Linda Diedhiou, Jian-Dong Huang, Alain Fischer, Geneviève de Saint Basile, Mickaël M. Ménager, Pablo Vargas, Fernando E. Sepulveda, Gaël Ménasché

**Affiliations:** ^1^ Université Paris Cité, Imagine Institute, Laboratory of Molecular Basis of Altered Immune Homeostasis, INSERM UMR1163, F-75015 Paris, France.; ^2^ Université Paris Cité, Imagine Institute, Laboratory of Single-Cell Inflammatory Responses and Multi-OMICs Networks, INSERM UMR1163, F-75015 Paris, France.; ^3^ Institute of Functional Genomics (IGF), CNRS UMR 5203, INSERM U1191, Université de Montpellier, Montpellier, France.; ^4^ Université Paris Cité, INSERM UMR1141, NeuroDiderot, Institut Hospitalo-Universitaire Robert-Debré du Cerveau de l’Enfant, Paris, France.; ^5^ Structure Fédérative de Recherche Necker, INSERM US24/CNRS UAR3633, Université Paris Cité, Institut Imagine, F-75015 Paris, France.; ^6^ School of Biomedical Sciences, Li Ka Shing Faculty of Medicine, The University of Hong Kong, Pokfulam, Hong Kong Special Administrative Region, China.; ^7^ Immunology and Pediatric Hematology Department, Necker Children’s Hospital, AP-HP, F-75015 Paris, France.; ^8^ Collège de France, F-75005 Paris, France.; ^9^ Leukomotion Lab, Paris Cité University, INSERM UMR-S1151, CNRS UMR-S8253, Institut Necker Enfants Malades, F-75015 Paris, France.; ^10^ Centre National de la Recherche Scientifique (CNRS), F-75015 Paris, France.

## Abstract

Dendritic cells (DCs) are professional antigen (Ag)–presenting cells that excel in initiating adaptive immune responses by continuously scanning peripheral tissues for Ags. To facilitate efficient DC migration, constant cross-talk between actin and microtubules is required to coordinate cytoskeletal networks and actomyosin contractility, but the related mechanisms have not been extensively characterized. We show that mouse DCs lacking Kif5b (the heavy chain of kinesin-1) exhibit a major impairment in cell migration in vivo and in vitro. Mechanistically, kinesin-1 coordinates cytoskeletal cross-talk between actin and microtubules during DC migration by modulating negatively RhoA activity through its interaction with GEF-H1, thereby limiting GEF-H1’s availability in the cytosol. The same mechanism operates in human primary monocyte–derived DCs and regulates efficient migration in a confined environment. Thus, our results highlight kinesin-1 as a key regulator of DC migration, through its coordinated control of cytoskeletal dynamics.

## Teaser

Kinesin-1 regulates DC migration by coordinating cytoskeletal cross-talk via the modulation of RhoA activity.

## INTRODUCTION

Dendritic cells (DCs) continuously scan peripheral tissues for danger signals and then migrate to draining lymph node (dLN) to initiate adaptive immune responses against pathogens or tumor cells by adopting an ameboid type of migration in which focal adhesions are not required (*1*, *2*). During their lifetime, DCs adopt different patterns of migration between the peripheral tissues and the lymphoid organs (*3*, *4*). Immature DCs sample their environment by internalizing extracellular material, allowing antigen (Ag) capture by phagocytosis and macropinocytosis (*5*–*7*). This Ag capture requires a slow migration, characterized by an enrichment of the Cdc42-Arp2/3–dependent actin pool at the front of the cells, which limits migration and promotes Ag capture (*8*). In the presence of a pathogen-associated molecular pattern or a damage-associated molecular pattern, the DCs rapidly commit to a maturation program that down-regulates Ag uptake and up-regulates the surface proteins controlling Ag presentation and migration to lymphoid tissues through lymphatic vessels [i.e., major histocompatibility complex class II (MHCII) and the CCR7 chemokine receptor] (*3*, *4*, *9*, *10*). The DCs then adopt a fast migration pattern, which is mediated by a RhoA-mDia1–dependent actin pool at the rear of the cells (*8*). Constant cross-talk between actin and microtubules is needed to facilitate efficient migration and locally coordinate cytoskeletal networks and actomyosin contractility (*11*). The small guanosine triphosphatase (GTPase) RhoA cycles between an active [guanosine triphosphate (GTP)–bound] and an inactive (guanosine diphosphate–bound] forms and is one of the key players in this cross-talk (*11*). Active RhoA then activates several downstream effectors such as Rho-associated protein kinase and the formin mDia1 to promote stress fiber formation, actin polymerization, and actomyosin-dependent contraction (*12*). RhoA activation is mediated by another important player in this process, the RhoA-specific exchange factor GEF-H1 (ARHGEF2, also known as Lfc in mice), which binds to polymerized microtubules (*13*–*15*). Upon microtubule depolymerization or acetylation, GEF-H1 is released from its sequestered state. This promotes RhoA activation, thereby establishing a direct links between microtubule dynamics and actomyosin networks (*14*, *16*, *17*). Furthermore, GEF-H1’s activity is down-regulated when sequestrated by the dynein motor light-chain protein Tctex-1 on microtubules (*18*). This mechanism illustrates the intricate interplay between different cytoskeletal components and their regulatory factors.

We have previously investigated the role of kinesin-1 (the archetypal member of the kinesin family), which mediates cargo transport to the plus-end of microtubules, in several types of immune cells (*19*–*23*). Kinesin-1 is a tetrameric protein constituted by two heavy chains (KIF5A, KIF5B, or KIF5C) and two light chains (KLC1, KLC2, KLC3, or KLC4) (*24*). The gene coding for the major heavy-chain isoform of kinesin-1 in murine DCs is *Kif5b* (*21*). Our recent work that used the conditional knockout mouse (cKO^Kif5b^, which lacks *Kif5b* in all cells of the hematopoietic lineage) demonstrated that kinesin-1 is involved in effective antitumor immune responses to melanoma (*21*). Mechanistically, we showed in vitro and in vivo that kinesin-1 regulates Ag cross-presentation by cDC1s to cognate cytotoxic T lymphocytes, by controlling early endosome maturation and thus intracellular Ag and MHCI trafficking (*21*).

Although DC migration involves two distinct actin nucleation machineries, the mechanisms that regulate the cross-talk between actin and microtubules during DC migration are not fully understood. Here, we leveraged cKO^Kif5b^ mice to show that kinesin-1 regulates efficient DC migration in vivo and in vitro. Our results show that kinesin-1 coordinates a cytoskeletal cross-talk between actin and microtubules in DCs undergoing ameboid migration by modulating negatively RhoA activity through its interaction with GEF-H1. We also found that in human primary monocyte–derived DCs (MDDCs), kinesin-1, microtubules, and modulation of RhoA activity are required for efficient migration in a confined environment.

## RESULTS

### Kinesin-1 expression modulates the transcription of genes involved in ameboid migration, chemotaxis, and actin reorganization in conventional splenic cDC1

**Fig. 1.**
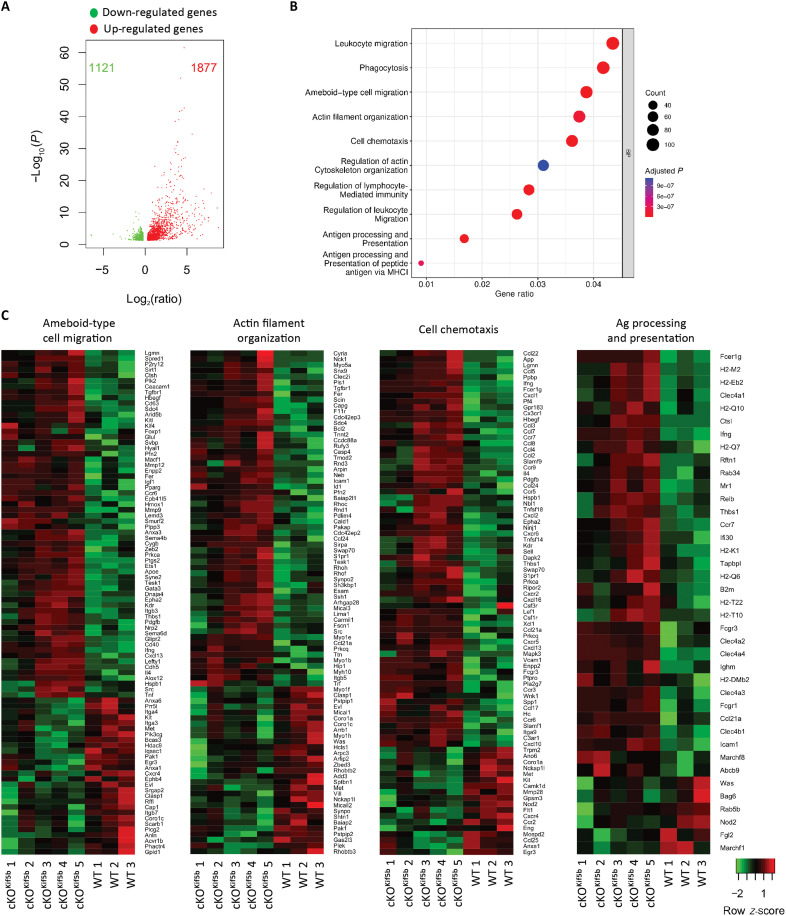
Kif5b expression in cDC1 modulates the transcriptional signatures related to ameboid migration, chemotaxis, and actin reorganization. (**A**) Volcano plot of DEGs (fold change ≥ 1.2; *P* < 0.05) in cDC1^Kif5b^ and control cDC1^WT^, showing the adjusted *P* value (−log_10_) versus fold change (log_2_). Up-regulated and down-regulated genes are shown in red and green, respectively. Total numbers in each group are indicated in red and green, respectively. (**B**) Dot plot of the GO BP terms enrichment analysis for DEGs. The dot color gradient represents the adjusted *P* value, while the size represents the gene count. Pathways enrichment analysis was performed using Ingenuity software. (**C**) Heatmaps of DEGs (fold change ≥ 1.2; *P* < 0.05) between cDC1^Kif5b^ and cDC1^WT^ involved in ameboid-type cell migration, actin filament organization, cell chemotaxis, and Ag processing and presentation. Three to five biological replicates are shown per group. The *z*-score represents gene up-regulation (red, positive values) or down-regulation (green, negative values) of genes.

To characterize the molecular networks modulated by the expression of kinesin-1 in DCs, we analyzed the transcriptome of conventional splenic DCs (cDC1) isolated from the spleen of control [wild-type (WT)] mice and cKO^Kif5b^ mice. We found that 2998 genes were differentially expressed in cDC1^Kif5b^ versus cDC1^WT^, 1121 genes were significantly down-regulated in cKO^Kif5b^ mice, and 1877 were significantly up-regulated (Fig. 1A). To determine the functional profile of these differentially expressed genes (DEGs), we analyzed the gene ontology (GO) biological process (BP) enrichment. The GO BP term analysis revealed an enrichment in pathways related to ameboid migration, cell chemotaxis, and actin reorganization (Fig. 1, B and C). In line with our previous report on kinesin-1’s role in regulating Ag presentation to T lymphocytes (*21*), we observed consistent enrichment in the GO BP terms related to Ag processing and presentation (Fig. 1, B and C). Overall, these results suggest that kinesin-1 regulate DC migration, chemotaxis, and actin reorganization in addition to Ag presentation.

### Kinesin-1 modulates DC migration and Ag capture

**Fig. 2.**
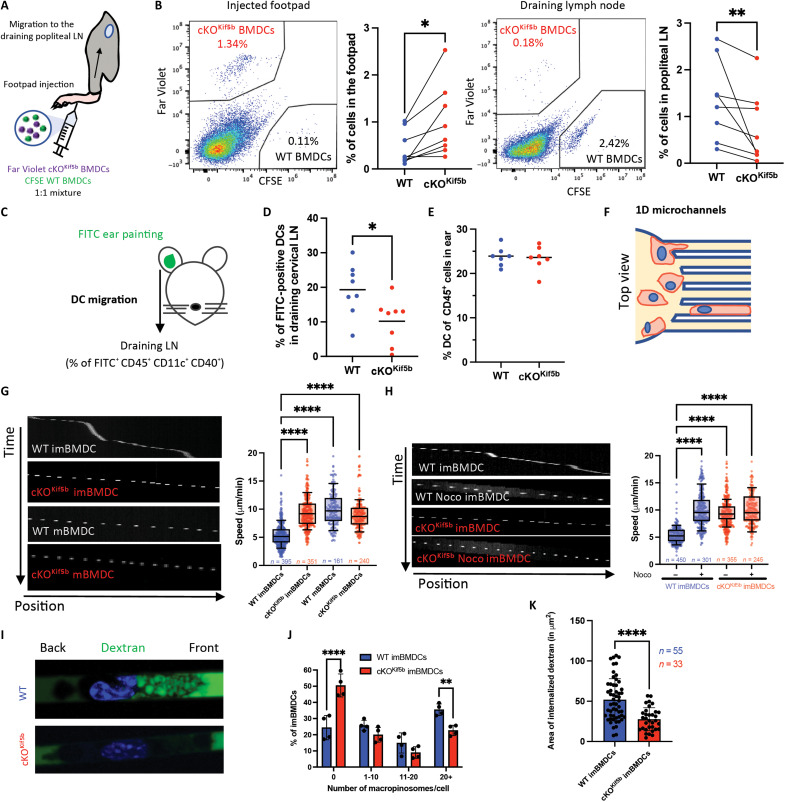
Kinesin-1 regulates DC migration in vivo and in vitro and Ag capture in vitro. (**A**) Graphical summary of the competitive homing assay. (**B**) Representative flow cytometry plots of WT and cKO^Kif5b^ BMDCs recovered from injected footpads and draining popliteal LNs 24 hours posttransfer. Graphs show the percentage of recovered WT and cKO^Kif5b^ BMDCs in footpads and LNs of WT recipients (*n* = 8). Statistical significance was determined in a Wilcoxon matched-pairs test (**P < 0.01; *P < 0.05). (**C**) Graphical summary of FITC ear painting. (**D**) Percentage of FITC^+^ DCs in draining LNs of WT and cKO^Kif5b^ mice (*n* = 8 per group). Statistical significance was determined in an unpaired *t* test (*P < 0.05). (**E**) Proportion of DCs among CD45^+^ cells in ears of WT and cKO^Kif5b^ mice (WT mice, *n* = 7; cKO^Kif5b^ mice, *n* = 7). (**F**) Top view of the one-dimensional (1D) microchannel assay. (**G**) Left: Kymographs of WT and cKO^Kif5b^ immature BMDCs (imBMDCs) and mature BMDCs (mBMDCs) in 8-μm-wide channels. Right: Mean migration speed of WT and cKO^Kif5b^ imBMDCs and mBMDCs, pooled from four independent experiments. Statistical significance was determined in a nonparametric one-way analysis of variance (ANOVA) with multiple-comparisons (Kruskal-Wallis) test (****P < 0.0001). (**H**) Left: Kymographs of WT and cKO^Kif5b^ imBMDCs and mBMDCs in 8-μm-diameter microchannels with or without nocodazole (Noco). Right: Quantification of mean speed, pooled from four independent experiments. Statistical significance was determined in a Kruskal-Wallis test (****P < 0.0001). (**I**) WT and cKO^Kif5b^ imBMDCs were seeded in 8-μm microchannels. After 16 hours, channels were filled with Alexa Fluor 488 (AF488)–dextran (10 kDa). Imaging was performed 1 hour later. Representative cell images are shown. (**J**) Distribution of macropinosome numbers per cell in WT and cKO^Kif5b^ BMDCs (*n* > 33 cells per condition, pooled from four independent experiments). Statistical significance was determined in a two-way ANOVA with Sidak’s correction (**P < 0.01; ****P < 0.0001). (**K**) Quantification of internalized dextran area in WT and cKO^Kif5b^ BMDCs. Means ± SD is shown. Statistical significance was determined in an unpaired *t* test (****P < 0.0001).

To evaluate the ability of DCs lacking Kif5b to migrate in a physiological context in vivo, we performed competitive homing assays in WT mice. Lipopolysaccharide (LPS)–activated bone marrow–derived DCs (BMDCs) obtained from WT mice and cKO^Kif5b^ mice and labeled with various fluorochromes were injected into the footpad (Fig. 2A). We used flow cytometry 24 hours after cell transfer to monitor the arrival of Kif5b-deficient BMDCs and WT BMDCs at the popliteal dLN. Fewer cKO^Kif5b^ BMDCs compared with WT BMDCs were recovered from the dLN (Fig. 2B and fig. S1). Accordingly, we collected more transferred cKO^Kif5b^ BMDCs than WT BMDCs from the footpad (Fig. 2B and fig. S1). These data suggest that DCs lacking Kif5b migrate less efficiently than WT DCs from the periphery to the LN and remain at the injection site. Next, to evaluate the ability of endogenous DCs lacking Kif5b to migrate from the skin to dLNs, we painted the skin from the ear of control mice and cKO^Kif5b^ mice with a fluorescein isothiocyanate (FITC)–containing irritant solution (Fig. 2C). Twenty-four hours later, the proportion of endogenous FITC-positive DCs in the draining LN was found to be lower in the cKO^Kif5b^ mice than in the WT mice (Fig. 2D). This difference could not be attributed to variations in the proportion of DCs in the ear of cKO^Kif5b^ mice because it was similar to that of control mice (Fig. 2E). Overall, these results demonstrate that kinesin-1 regulates efficient DC migration in vivo.

To further explore the role of kinesin-1 in DC migration in vitro, we next used microfabricated channels to study the ability of DCs lacking Kif5b to migrate in a confined environment (microchannels; Fig. 2F) (*25*). We compared the spontaneous motility in microchannels of immature BMDCs (imBMDCs) and LPS-activated (mature) BMDCs (mBMDCs) obtained from control mice and cKO^Kif5b^ mice. The results showed that the lack of Kif5b disrupted the normal motility pattern of imBMDCs (i.e., an alternation of slow and fast migration phases) (*25*). Kif5b-deficient imBMDCs lacked slow motility phases during migration and therefore exhibited a higher mean speed than their control counterparts (Fig. 2G, fig. S2, and movies S1 and S2). This phenotype was observed for different levels of confinement (i.e., both 4- and 8-μm-wide microchannels) and adhesivity [i.e., both fibronectin-coated and polyethylene glycol (PEG)–coated channels surfaces] (Fig. 2G, fig. S2, and movies S1 and S2). Kif5b-deficient imBMDCs reached much the same speed as LPS-activated mBMDCs (Fig. 2G and movies S1 to S4). Differences in cell motility between control cells and Kif5b-deficient cells were not related to spontaneous DC activation in the absence of Kif5b (fig. S3).

Given that kinesin-1 is a microtubule-based motor, we also investigated the role of microtubules in the spontaneous motility of BMDCs. We found that disruption of the microtubule network (by nocodazole) in WT imBMDCs abolished the slow motility phases, whereas it did not modify cell activation, similar to the phenotype observed in untreated Kif5b-deficient imBMDCs (Fig. 2H and fig. S3). Nocodazole treatment did not affect the motility of Kif5b-deficient mBMDCs (Fig. 2H). These results indicate that both kinesin-1 and microtubules are required for establishing slow migration phases during the ameboid migration of imBMDCs.

Given that fast DC motility decreases Ag uptake (*4*), we next looked at whether kinesin-1 plays a role in Ag uptake during confined migration. To visualize Ag uptake by macropinosomes, we filled microchannels with fluorescently labeled dextran. We observed that nearly 50% of the Kif5b-deficient imBMDCs versus 20% of control cells imBMDCs did not internalize dextran in the experimental setting used (Fig. 2, I and J). Furthermore, the area of macropinosomes internalized by Kif5b-deficient imBMDCs was significantly smaller than that in control cells (Fig. 2K). Considering that our previous work had not evidenced a role of kinesin-1 in the uptake of soluble or particulate Ags in a nonconfined environment, our present results suggest that kinesin-1 indirectly regulates Ag sampling by controlling imBMDC motility (*21*).

**Fig. 3.**
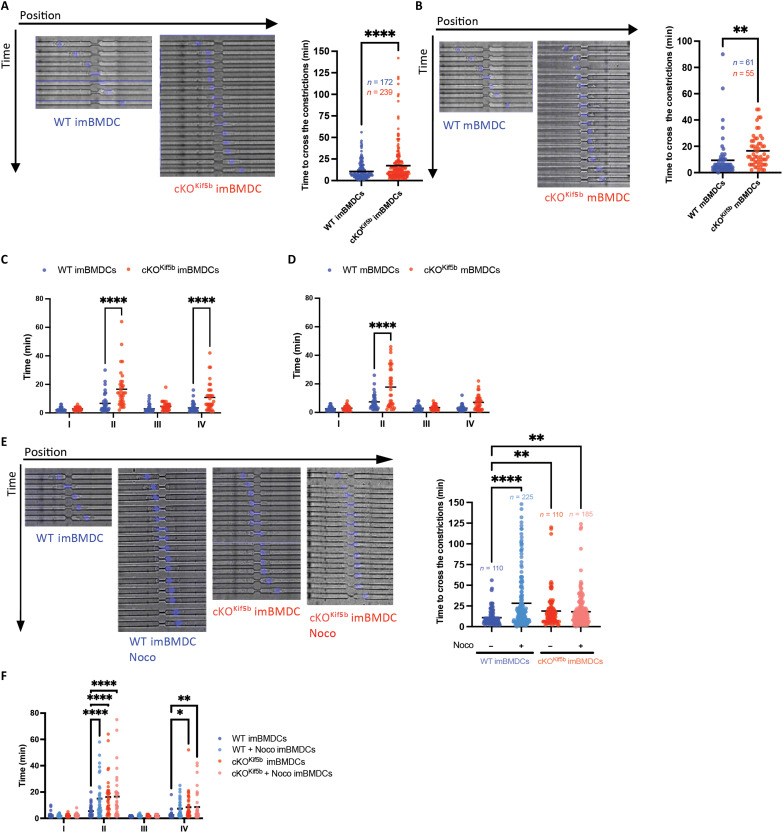
Kinesin-1 is required for DC passage through constrictions. (**A** and **B**) Left: Representative time lapse images of WT and cKO^Kif5b^ imBMDCs (A) and mBMDCs (B) stained with Hoechst reagent (DNA; blue) and passing through a 1.5-μm constriction. Right: Quantification of the constriction passage time. The number of cells (*n*) is indicated for each condition, pooled from *N* = 5 independent experiments for imBMDCs and *N* = 2 independent experiments for mBMDCs. Statistical significance was determined by an unpaired *t* test (****P < 0.0001; **P < 0.01). (**C** and **D**) Quantification of the time spent by WT and cKO^Kif5b^ imBMDCs and mBMDCs during each of the four transmigration phases: (I) cell front entry into the constriction, (II) nuclear passage, (III) nuclear exit, and (IV) cell exit. Statistical significance was determined in a two-way ANOVA, using Sidak’s correction (****P < 0.0001). (**E**) Left: Representative time lapse images of WT and cKO^Kif5b^ imBMDCs treated (or untreated) with nocodazole stained with Hoechst reagent (DNA; blue), passing through a 1.5-μm constriction. Right: Quantification of the constriction passage time. The number of cells (*n*) is indicated for each condition, pooled from *N* = 2 independent experiments. Statistical significance was determined in a nonparametric one-way ANOVA with multiple comparisons (Kruskal-Wallis) (****P < 0.0001; **P < 0.01). (**F**) Quantification of the time spend by WT and cKO^Kif5b^ imBMDCs treated (or untreated) with nocodazole during each of the four phases of transmigration: (I) cell front entry into the constriction, (II) nuclear passage, (III) nuclear exit, and (IV) cell exit. Statistical significance was determined in a two-way ANOVA using Sidak’s correction (****P < 0.0001; **P < 0.01).

Notably, our data show that Kif5b-deficient DCs have opposite behaviors when migrating in complex environments that require strong cell deformation (i.e., in vivo) versus obstacle-free microchannels. Given that (i) the nucleus is the stiffest organelle in the cell and (ii) nuclear deformation determines migration ability in three-dimensional environments, we investigated the impact of Kif5b deficiency on DC ability to deform the nucleus and migrate through microchannels with constrictions (8-μm microchannels containing constrictions of 1.5 μm in width and 15 μm in length). Time-lapse imaging showed that imBMDCs and mBMDCs lacking Kif5b took significantly longer than control cells to pass through 1.5-μm-width constrictions (Fig. 3, A and B, and movies S5 to S8). With regard to the four phases of transmigration [cell front entry into a constriction, nuclear passage, nuclear exit, and cell exit as demonstrated in (*26*)], it was the nuclear passage that was the step most affected by the kinesin-1 defect, independently of the BMDC activation status (Fig. 3, C and D). The cell exit phase was also affected for Kif5b-deficient imBMDCs (Fig. 3C). In line with our previous observations, microtubule depletion in WT imBMDCs recapitulated the Kif5b-deficient imBMDC phenotype. The cells took longer to pass through the constriction because the nuclear passage time was extended (Fig. 3, E and F). In contrast, nocodazole treatment of Kif5b-deficient imBMDCs had no effect on their overall passage time nor on the durations of the various transmigration phases (Fig. 3, E and F). These findings indicate that kinesin-1 and microtubules coordinate nuclear deformation when a DC migrates through micrometric spaces. Overall, these data demonstrate that kinesin-1 regulates DC migration (both in vivo and in vitro), nuclear deformation, and Ag uptake in a confined environment.

### Kinesin-1 is not required for DC chemotaxis

**Fig. 4.**
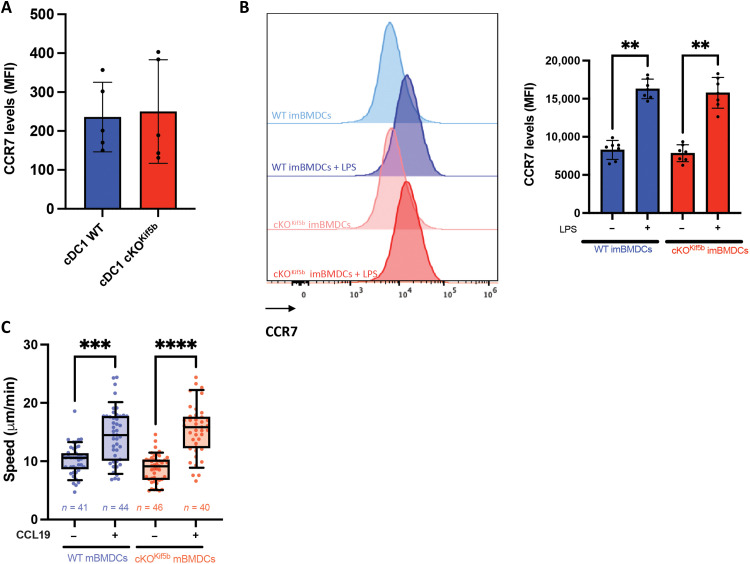
Kif5b does not play a role in regulating chemokine responsiveness. (**A**) CCR7 level from cDC1 isolated from the spleen of WT mice and cKO^Kif5b^ mice (WT mice, *n* = 5; cKO^Kif5b^ mice, *n* = 5). (**B**) Left: Representative flow cytometry quantification of CCR7 in WT and cKO^Kif5b^ imBMDCs treated (or untreated) with LPS. Right: CCR7 levels from WT and cKO^Kif5b^ imBMDCs treated (or untreated) with LPS is shown (*N* = 4 independent experiments). Statistical significance was determined in a nonparametric one-way ANOVA with multiple comparisons (Kruskal-Wallis) (**P < 0.01). (**C**) Quantification of the mean migratory speed of mature WT and cKO^Kif5b^ BMDCs within microchannel, with or without the presence of CCL19 on the opposite side of the cells. The number of cells (*n*) is indicated for each condition, pooled from *N* = 2 independent experiments. Statistical significance was determined in a nonparametric one-way ANOVA with multiple comparisons (Kruskal-Wallis) (****P < 0.0001; ***P < 0.001).

Given that CCR7 was found to be differentially expressed in splenic cDC1^Kif5b^ compared to cDC1^WT^ (Fig. 1C), we evaluated CCR7 protein levels in both cDC1s and BMDCs. cDC1^Kif5b^ exhibited normal CCR7 expression at the plasma membrane compared to cDC1^WT^ (Fig. 4A). Likewise, Kif5b-deficient BMDCs up-regulated CCR7 in response to LPS stimulation to a degree comparable to that of WT BMDCs (Fig. 4B). To further assess the functional responsiveness of Kif5b-deficient BMDCs to chemokines, we introduced CCL19 into the microchannels. Upon CCL19 exposure, both control and Kif5b-deficient mBMDCs presented with similar increased in speed (Fig. 4C). Collectively, these finding indicate that kinesin-1 does not play a role in regulating CCR7 response.

### Kinesin-1 motility is related to the RhoA pathway

**Fig. 5.**
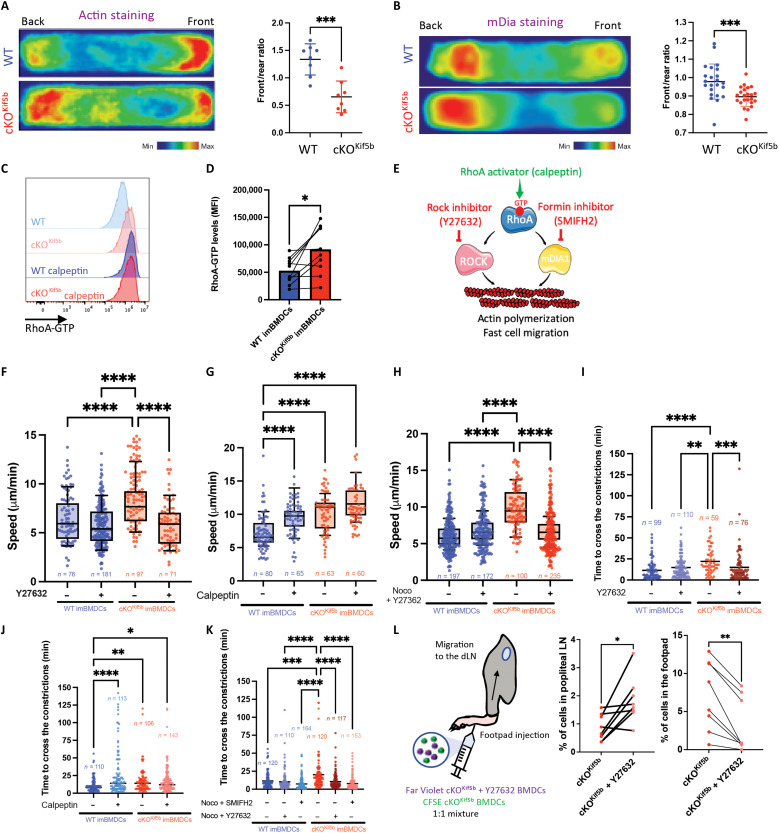
Kif5b deficiency promotes RhoA activation. (**A** and **B**) Left: Actin-phalloidin (A) and mDia1 (B) density maps of WT and cKO^Kif5b^ imBMDCs migrating in 8-μm microchannels (*n* > 48 cells per condition, pooled from *N* = 2 independent experiments). Right: Quantification of the front/rear ratio of the signal intensity is shown. Statistical significance was determined in an unpaired *t* test (***P < 0.001). (**C**) Flow cytometry quantification of RhoA-GTP in WT and cKO^Kif5b^ imBMDCs treated (or untreated) with calpeptin. (**D**) RhoA-GTP levels from WT and cKO^Kif5b^ imBMDCs is shown (*N* = 9 independent experiments). Statistical significance was determined using Wilcoxon matched-pairs test (*P < 0.05). (**E**) Schematic representation of different signaling pathways related to RhoA activation, including the drugs used. Created using Servier Medical Art; licensed under CC BY 4.0 (https://creativecommons.org/licenses/by/4.0/). (**F** to **H**) Quantification of the mean speed for immature WT and cKO^Kif5b^ BMDCs treated (or untreated) with Y27632 (F), calpeptin (G), and nocodazole plus Y27632 (H) in 8-μm microchannels. The number of cells (*n*) is indicated for each condition, pooled from *N* = 2 or 3 independent experiments. Statistical significance was determined in a nonparametric one-way ANOVA with multiple comparisons (Kruskal-Wallis) (****P < 0.0001). (**I** to **K**) Quantification of the time to pass through 1.5-μm constrictions for WT and cKO^Kif5b^ imBMDCs treated (or untreated) with Y27632 (I), calpeptin (J), and nocodazole plus Y27632 or SMIFH2 (K). The number of cells (*n*) is indicated for each condition, pooled from *N* = 2 independent experiments for each condition. Statistical significance was determined in a nonparametric one-way ANOVA with multiple comparisons (Kruskal-Wallis) (****P < 0.0001; ***P < 0.001; **P < 0.01; *P < 0.05). (**L**) Left: Graphical summary of the competitive homing assays. Right: Quantification of recovered cKO^Kif5b^ BMDCs and cKO^Kif5b^ BMDCs treated with Y27632 in injected footpad and draining popliteal LN (WT recipient mice, *n* = 8). Statistical significance was determined using Wilcoxon matched-pairs test (**P < 0.01; *P < 0.05).

Two actin pools have been shown to regulate the biphasic migration of immature DCs (*8*). Because kinesin-1 modulates the transcriptional signatures of actin reorganization (Fig. 1, B and C) and is required for slow-phase motility (Fig. 2G), we investigated the distributions of the actin pools and the formin mDia1 in imBMDCs lacking Kif5b during confined migration. We observed an inversion of the front/rear ratio of actin pools and mDia1, with a significant enrichment at the rear of Kif5b-deficient imBMDCs versus WT imBMDCs (Fig. 5, A and B). We next assessed the activation status of the small GTPase RhoA, which is required for polymerization of the actin pool at the cell rear (*8*). By using an antibody that specifically binds to active RhoA, we showed that resting imBMDCs lacking Kif5b had a significantly higher basal level of RhoA-GTP than control cells (Fig. 5, C and D). The RhoA-GTP level in the absence of Kif5b was similar to that observed in WT or cKO^Kif5b^ imBMDCs treated with the RhoA activator calpeptin (Fig. 5, C and E). These results indicate that the absence of kinesin-1 in immature DCs is correlated with increased RhoA activation.

To determine whether the impact of kinesin-1 deficiency on DC migration was dependent on elevated RhoA-mediated signaling, we tested the effect of RhoA-modulating drugs, including the Rock inhibitor Y27632, the formin inhibitor SMIFH2, and the RhoA activator calpeptin (Fig. 5E). Both Y27632 and SMIFH2 decreased the speed of Kif5b-deficient imBMDCs and enabled cells to adopt a slow-phase migration pattern at much the same speed as WT imBMDCs (Fig. 5F, fig. S4A, and movie S9).

Conversely, calpeptin treatment of WT imBMDCs reproduced the phenotype of Kif5b-deficient imBMDCs (i.e., high speed and absence of slow migration phases) (Fig. 5G). The combination of nocodazole with a Rock or formin inhibitor restored the slow-phase motility of Kif5b-deficient imBMDCs, which suggests that microtubule depolymerization acts upstream of the RhoA pathway (Fig. 5H and fig. S4B). None of the three drugs induced the spontaneous activation of imBMDCs from WT or cKO^Kif5b^ mice (fig. S3).

Similarly, inhibition of RhoA activation by treatment with Y27632 or SMIFH2 restored the constriction passage time of Kif5b-deficient imBMDCs, which was similar to that observed for WT imBMDCs (Fig. 5I, fig. S4C, and movie S10). Conversely, activation of the RhoA pathway by calpeptin treatment increased the constriction passage time of WT imBMDCs but not Kif5b-deficient imBMDCs (Fig. 5J). The combination of nocodazole with a Rock or formin inhibitor also restored the constriction passage time of Kif5b-deficient imBMDCs (Fig. 5K). This finding indicates that hyperactivation of the RhoA pathway disrupts the DC’s nuclear deformation ability during migration.

To determine whether modulation of the RhoA pathway activity could restore a normal pattern of migration in DCs lacking Kif5b in vivo, we performed competitive homing assays in WT mice by injecting Kif5b-deficient mBMDCs treated or untreated with Rock inhibitor Y27632 into the footpad. Notably, Y27632-treated cKO^Kif5b^ BMDCs migrated more efficiently from the footpad to the dLN than untreated cKO^Kif5b^ BMDCs did (Fig. 5L). Thus, a reduction in RhoA pathway activity in DCs lacking Kif5b corrected the in vivo migration defect.

Given recent evidence that different actin pools are tightly interconnected (*27*), we evaluated the activation status of Cdc42 in Kif5b-deficient imBMDCs. Using an antibody specific for active Cdc42, we showed that the levels of Cdc42-GTP in Kif5b-deficient imBMDCs were comparable to those in control cells at the steady state and upon treatment with a Cdc42 activator (fig. S5A). To assess whether the role of kinesin-1 on DC migration was dependent on Cdc42-mediated signaling, we tested the impact of the Cdc42 inhibitor MBQ167 on migration in microchannels. Treatment with MBQ167 in WT BMDCs abolished slow motility phases, resulting in a higher mean speed compared to untreated WT cells (fig. S5B). Notably, MBQ167 treatment did not affect the mobility of Kif5b-deficient imBMDCs (fig. S5B). These results suggest that the kinesin-1 deficiency does not alter Cdc42-mediated signaling. Overall, our results highlight a key role for kinesin-1 in mediating the interplay between actin and microtubule networks, thereby promoting efficient DC migration both in vitro and in vivo through modulation of the RhoA pathway.

### Kinesin-1 modulates RhoA activation via GEF-H1

**Fig. 6.**
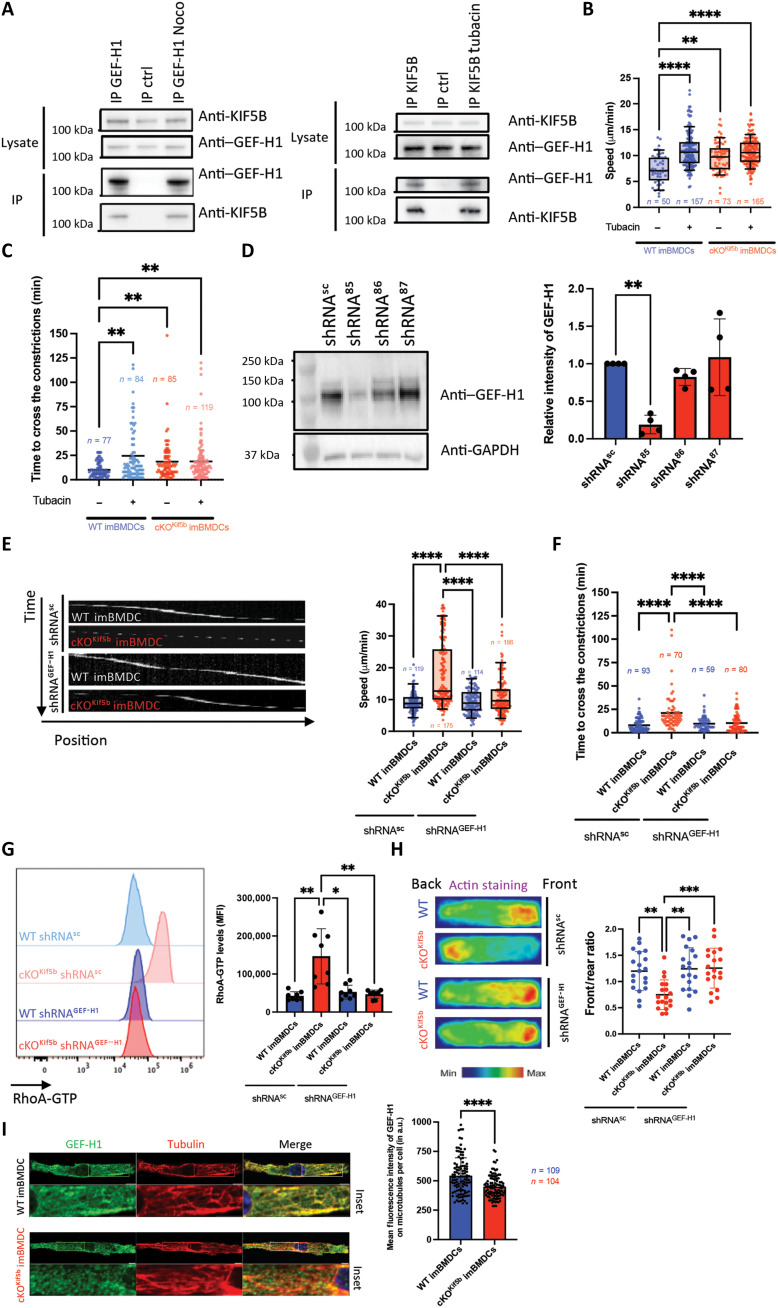
Kinesin-1 modulates RhoA activity via interaction with GEF-H1. (**A**) WT BMDCs treated (or untreated) with nocodazole or tubacin were lysed and immunoprecipitated (IP) with either isotype control, anti-GEF-H1, or anti-Kif5b antibodies. Immunoblots were analyzed for GEF-H1 and Kif5b (*N* = 4 experiments). (**B**) Mean migration speed of immature WT and cKO^Kif5b^ BMDCs, treated (or untreated) with tubacin, within 8-μm microchannels, pooled from two independent experiments. Nonparametric one-way ANOVA with multiple comparisons Kruskal-Wallis (****P < 0.0001; **P < 0.01). (**C**) Time required to pass 1.5-μm constrictions for WT and cKO^Kif5b^ imBMDCs, treated or untreated with tubacin, pooled from two independent experiments. Kruskal-Wallis (**P < 0.01). (**D**) Left: WT imBMDCs were infected with shRNAs targeting GEF-H1 (shRNA^85^, shRNA^86^, and shRNA^87^) or control (shRNA^sc^), selected, and lysed for Western blot [anti-GEF-H1 and anti–glyceraldehyde-3-phosphate dehydrogenase (GAPDH)], *N* = 4 independent experiments. Right: Quantification of the relative intensity of GEF-H1. Kruskal-Wallis (**P < 0.01). (**E**) Left: Kymographs of WT/cKO^Kif5b^ imBMDCs infected with shRNA^GEF-H1^ or control (shRNA^sc^) migrating in 8-μm microchannels. Right: Mean speed quantification (*N* = 4 experiments). Kruskal-Wallis (****P < 0.0001). (**F**) Time to cross 1.5-μm constrictions for WT/cKO^Kif5b^ imBMDCs with shRNA^GEF-H1^/shRNA^sc^ (*N* = 3 experiments). Kruskal-Wallis (****P < 0.0001). (**G**) Left: Flow cytometry quantification of RhoA-GTP levels in WT/cKO^Kif5b^ imBMDCs infected with shRNA^GEF-H1^ or shRNA^sc^. Right: RhoA-GTP level quantification (*N* = 3 independent experiments). Kruskal-Wallis (*P < 0.05; **P < 0.01). (**H**) Left: Actin-phalloidin density maps of WT and cKO^Kif5b^ imBMDCs infected with shRNA^GEF-H1^ or shRNA^sc^ (*n* > 28 cells per condition, pooled from *N* = 2 experiments) in 8-μm microchannels. Right: Quantification of the front/rear ratio of the signal intensity. Kruskal-Wallis (***P < 0.001; **P < 0.01). (**I**) Left: Immunofluorescence imaging of microtubule-associated GEF-H1 in WT/cKO^Kif5b^ imBMDCs (*n* > 104, *N* = 3). Boxed regions are displayed at higher magnification in the insets. Scale bars: 5 μm. Quantification on the mean fluorescence intensity (MFI) of microtubule-associated GEF-H1 per cell is shown of the right panel. Unpaired *t* test (****P < 0.0001). a.u., arbitrary units.

To characterize the molecular link between kinesin-1 and the RhoA pathway, we focused on the microtubule-associated RhoA-specific exchange factor GEF-H1. This factor reportedly mediates the cross-talk between microtubules and actin (*13*). Given that the dynein motor light-chain protein Tctex-1 interacts with GEF-H1 and modulates its activity (*18*), we investigated whether kinesin-1 and GEF-H1 interacted in DCs by performing coimmunoprecipitation assays with anti-GEF-H1 or anti-KIF5B. The results demonstrated that endogenous GEF-H1 forms a complex with Kif5b, as evidenced by their coimmunoprecipitation in BMDCs (Fig. 6A). Furthermore, treatment with either nocodazole or tubacin (an inducer of tubulin acetylation that promote GEF-H1 release from microtubules) had no effect on Kif5b/GEF-H1 complex formation, indicating that the interaction between GEF-H1 and Kif5b is independent of (i) the kinesin-1 interaction with microtubules and (ii) the pool of GEF-H1 that interacts directly with microtubules (Fig. 6A). Next, to establish how the modulation of GEF-H1’s interaction with microtubules influences DC migration in a confined environment, we treated WT and cKO^Kif5b^ BMDCs with tubacin. Tubacin treatment led to a higher speed in WT imBMDCs, which became similar to that of Kif5b-deficient imBMDCs, while BMDC activation was not modified (Fig. 6B and fig. S3). Similarly, tubacin treatment increased the time required for WT imBMDCs to pass through 1.5-μm constrictions, whereas imBMDCs lacking Kif5b were not affected (Fig. 6C). To investigate the role of GEF-H1 in DC motility, we knocked down GEF-H1 using short hairpin RNAs (shRNAs) and examined their effect on WT and cKO^Kif5b^ BMDCs. The most effective GEF-H1 knockdown was confirmed by Western blotting for shRNA^85^, which was, thus, subsequently used for cell migration experiments (Fig. 6D). GEF-H1 knockdown did not trigger spontaneous activation of WT or cKO^Kif5b^ BMDCs (fig. S6A). GEF-H1 knockdown restored slow phase migration and thus decreased the overall speed of Kif5b-deficient imBMDCs, which was similar to that of WT imBMDCs infected with scrambled shRNA or GEF-H1 shRNA (Fig. 6E and movies S11 to S14). Similarly, GEF-H1 knockdown restored the constriction passage time of infected Kif5b-deficient imBMDCs, which was then similar to that of WT imBMDCs infected with scrambled shRNA or GEF-H1 shRNA (Fig. 6F). Likewise, GEF-H1 knockdown in cKO^Kif5b^ BMDCs restored the RhoA-GTP level and the front/rear distribution of actin pools, i.e., to values observed for WT BMDCs infected with scrambled shRNA or GEF-H1 shRNA (Fig. 6, G and H). We next evaluated GEF-H1 localization in Kif5b-deficient imBMDCs under confined migration conditions. Antibody specificity was validated in WT imBMDCs infected with either scrambled shRNA or GEF-H1 shRNA (fig. S6B). Control cells (WT imBMDC shRNA^SC^) showed robust colocalization of GEF-H1 with microtubule filaments (fig. S6B). Notably, Kif5b-deficient imBMDCs exhibit significantly reduced GEF-H1 microtubule–binding capacity (Fig. 6I).

Overall, these results highlight GEF-H1 as a previously unidentified component of the Kif5b interactome, which mediates cross-talk between the microtubule motor kinesin-1 and RhoA activity. When released into the cytosol, GEF-H1 might act as a molecular rheostat by fine-tuning RhoA-driven actin polymerization during DC migration in a confined environment.

### The roles of kinesin-1, microtubules, and RhoA activity in human DC migration

**Fig. 7.**
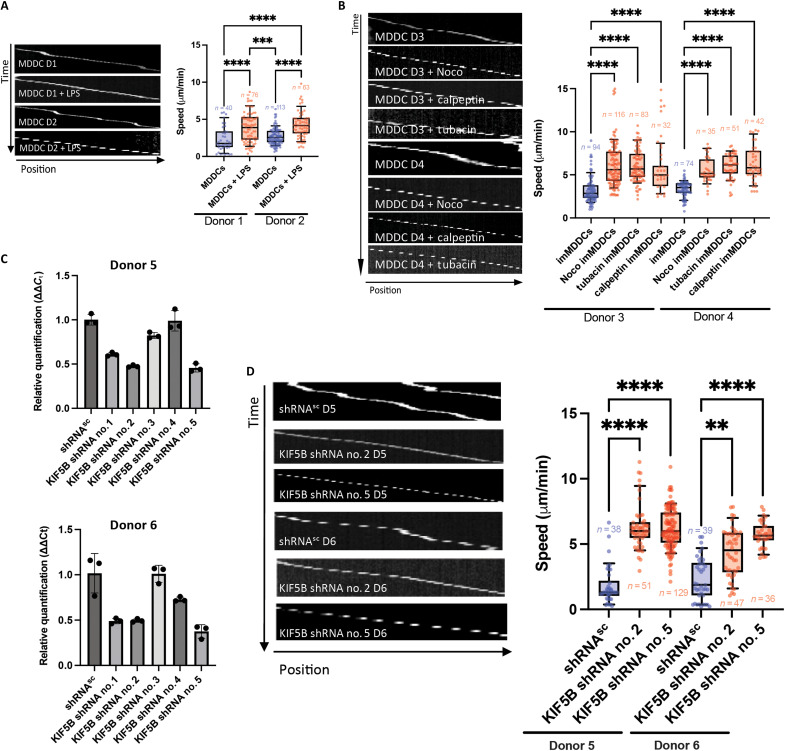
Kinesin-1, microtubules, and modulation of RhoA activity are required for efficient MDDC migration in a confined environment. (**A**) Left: Representative kymographs are shown for MDDCs from two donors (D1 and D2) treated (or untreated) with LPS and migrating in 8-μm microchannels. Right: Quantification of the mean speed of MDDCs treated (or untreated) with LPS. The number of cells (*n*) is indicated for each condition. Statistical significance was determined in a nonparametric one-way ANOVA with multiple comparisons (Kruskal-Wallis) (****P < 0.0001; ***P < 0.001). (**B**) Left: Representative kymographs are shown for MDDCs from two donors (D3 and D4) treated (or untreated) with nocodazole, or calpeptin, or tubacin and migrating in 8-μm-diameter microchannels. Right: Quantification of the mean speed of MDDCs treated (or untreated) with nocodazole, calpeptin, or tubacin. The number of cells (*n*) is indicated for each condition. Statistical significance was determined in a nonparametric one-way ANOVA with multiple comparisons (Kruskal-Wallis) (****P < 0.0001). (**C**) MDDCs from two donors (D5 and D6) were infected with viral particles expressing with one of five shRNA targeting KIF5B or with control shRNA (shRNA^sc^). KIF5B transcript levels were determined in a real-time polymerase chain reaction (PCR) assay. Representative results from one of three experiments are shown. (**D**) Left: Representative kymographs are shown for MDDCs from two donors (D5 and D6) infected with one of two selected shRNAs targeting KIF5B or with control shRNA (shRNA^sc^) and migrating in 8-μm microchannels. Right: Quantification of the mean speed of the infected MDDCs. The number of cells (*n*) is indicated for each condition. Statistical significance was determined in a nonparametric one-way ANOVA with multiple comparisons (Kruskal-Wallis) (****P < 0.0001; **P < 0.01).

We also used microchannels to characterize the roles of kinesin-1, the RhoA pathway, and the microtubule network in the migratory ability of human MDDCs. Spontaneous motility of immature MDDCs in the microchannel alternated between slow and fast migration, as described for mouse BMDCs (Fig. 7A, fig. S7A, and movie S15). Similarly, LPS-activated MDDCs migrated faster because, as expected, slow motility phases were absent (Fig. 7A, fig. S7A, and movie S16). Immature MDDCs treated with nocodazole, calpeptin, or tubacin were no longer able to adopt slow migration phases, suggesting that an increase in RhoA activity in human immature DCs is also associated with a higher migration speed (Fig. 7B and movies S17 to S19). None of the drugs induced an activation of MDDCs (fig. S7A).

Given that *KIF5B* is the only kinesin-1 heavy chain expressed in the human hematopoietic lineage (including DCs) (*23*), we next assessed the functional consequences of inhibiting kinesin-1 expression on MDDCs using shRNA to inactivate KIF5B. We selected the two shRNAs (*KIF5B* shRNA nos. 2 and 5) that had the most potent inhibitory activity on KIF5B expression in cells from several donors (Fig. 7C and fig. S8A) and confirmed the absence of spontaneous MDDC activation by KIF5B knockdown (fig. S7B). We then tested the ability of infected MDDCs to migrate in microchannels. KIF5B knockdown in immature MDDCs mimicked what was observed in the mouse Kif5b-deficient BMDCs, i.e., a higher average speed due to the absence of slow motility phases (Fig. 7D, fig. S8B, and movies S20 and S21). Overall, these results reveal that kinesin-1, microtubules, and modulation of RhoA are also required for efficient human DC migration in a confined environment.

## DISCUSSION

Cell motility is essential for DC functions, such as continuously scanning peripheral tissues for danger signals and then returning to lymphoid organs to initiate adaptive immune responses against pathogens and tumor cells. Although DC migration relies strongly on actin nucleation machineries, the mechanisms that regulate cross-talk between actin and microtubules during this process have not been extensively characterized. The present study provided in vivo and in vitro evidence to show that the Kif5b heavy-chain isoform of kinesin-1 controls DC migration in humans and mice.

**Fig. 8.**
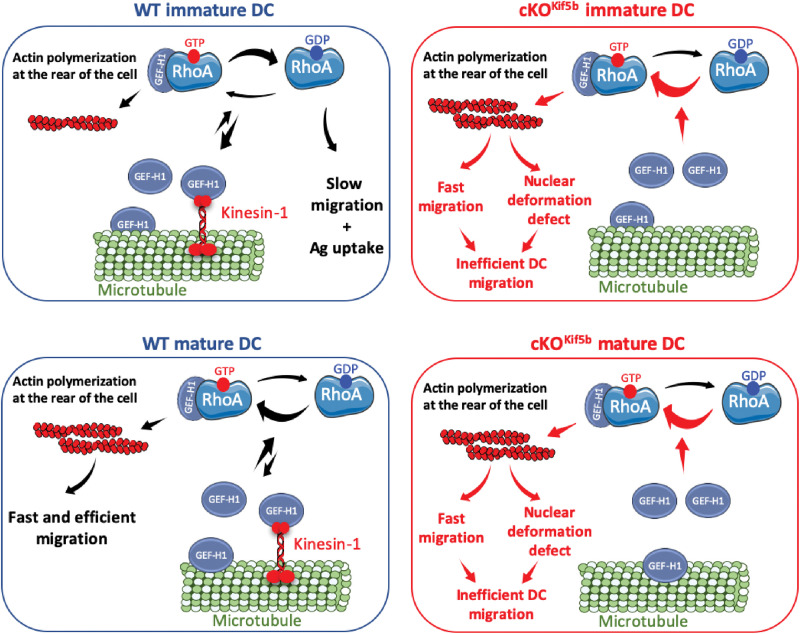
A model of the role of kinesin-1 in the cross-talk between actin and microtubule cytoskeletons, promoting efficient ameboid DC migration. In immature DCs, kinesin-1 coordinates the cytoskeletal cross-talk between actin and microtubules by modulating negatively RhoA activity through its interaction with GEF-H1. This interplay is required to establish slow migration phases and facilitate Ag uptake. In immature DCs that lack kinesin-1, unbound GEF-H1 leads to inappropriate hyperactivation of the RhoA pathway, resulting in (i) increased polymerization of the actin pool at the rear of the cell, which promoted fast migration; (ii) altered nuclear deformation; and (iii) reduced capacity for Ag uptake. In mature DCs, the cross-talk between microtubules and actin is mediated by fine-tuned modulation of RhoA activity, enabling efficient ameboid migration in complex environments. The absence of kinesin-1 in mature DCs similarly results in uncontrolled activation of RhoA due to the free pool of GEF-H1, leading to inefficient ameboid migration. Created using Servier Medical Art; licensed under CC BY 4.0 (https://creativecommons.org/licenses/by/4.0/).

A lack of Kif5b (i) modulated the transcription of genes involved in ameboid migration, cell chemotaxis, and actin reorganization in cDC1s; (ii) impaired DC migration in vivo and in vitro by altering spontaneous motility, nuclear deformation, and Ag uptake; and (iii) interfered with the cross-talk between actin and microtubule via modulation of the RhoA activity. Our experiments with human MDDCs also showed that KIF5B, microtubules, and the modulation of RhoA activity are required for efficient migration in a confined environment. Mechanistically, kinesin-1 appears to coordinate cytoskeletal interplay during DC migration by negatively modulating RhoA activity via its interaction with GEF-H1 (Fig. 8 and fig. S9). In contrast, chemokine responsiveness was found to be independent of Kif5b.

We were surprised to find such a significant difference in DEGs between cDC1^Kif5b^ and cDC1^WT^, potentially revealing an unexpected role for kinesin-1 in the regulation of gene transcription. Supporting this notion, a recent study demonstrated that certain kinesins, including kinesin-1, have LxxLL motifs enabling them to interact with nuclear receptors and regulate transcription (*28*). Although this study primarily focused on KIF1B, it suggests a broader role for kinesins in nuclear receptor–mediated transcriptional regulation. Further research will be necessary to thoroughly investigate the potential role of kinesin-1 in transcriptomic regulation, contributing to the understanding of cytoskeletal protein functions beyond their traditional roles.

In a previous study, we showed that kinesin-1 regulated early endosome dynamics in DCs by controlling the scission of endosomal tubulations that allows Ag trafficking and MHCI recycling (*21*). A defect in kinesin-1–based early endosome transport leads to a marked impairment in Ag cross-presentation and thus a poor antitumor response in vivo (*21*). Our present data show that the regulation of DC migration depends on a complex formation between kinesin-1 and the RhoA-specific exchange factor, GEF-H1. Tubacin treatment of WT DCs (which forces the release of GEF-H1 from acetylated microtubules and triggers RhoA activation) phenocopied the motility impairment observed in Kif5b-deficient DCs (i.e., the absence of slow motility phases and a longer time required for passage through micrometric constrictions) (fig. S9). Furthermore, knockdown of GEF-H1 in Kif5b-deficient DCs restored the slow migration phases in a confined environment and the usual speed for constriction passage, reversed the front/rear ratio of the actin pools, and decreased the level of RhoA-GTP level. The interaction between Kif5b and GEF-H1 is expected to repress GEF-H1’s nucleotide exchange activity, as has been demonstrated for the interaction between the dynein light chain, Tctex-1 and GEF-H1 (*18*). Thus, our data identified kinesin-1 as a key player in the modulation of RhoA activity during DC migration through changes in GEF-H1’s accessibility by limiting its cytosolic localization and therefore its activity. In the future, it would be interesting to characterize the domains of each partner involved in this interaction, which would confirm its direct nature and provide mechanistic information about the binding process. The generation of Kif5b or GEF-H1 mutants with altered protein interactions should also provide a better understanding of specific functions in the cross-talk between actin and microtubules during DC migration.

We found that nocodazole-induced microtubule disassembly in WT DCs impaired the ability to adopt slow-phase migration and to pass constrictions rapidly but did not suppress the ability to migrate in microchannels. This is expected because microtubules are not involved in generating the forces that govern ameboid cell migration (*13*). In contrast, it is well known that microtubule depolymerization can activate RhoA by increasing the amount of active GEF-H1, thereby coordinating actin polymerization and contractility with changes in microtubule dynamics (*13*). It was shown recently that microtubule disassembly in migrating DCs coordinates protrusion retraction and actomyosin contraction through the local release of GEF-H1, which is essential for maintaining a coherent cell shape (*14*). While our study and that of Kopf *et al.* (*14*) identify microtubule disruption as a key driver of impaired DC migration in confined environments, our work goes further by elucidating a specific molecular mechanism involving kinesin-1. More specifically, we demonstrate that kinesin-1 orchestrates cross-talk between actin and microtubules by negatively modulating RhoA activity through its interaction with GEF-H1, thereby limiting GEF-H1’s accessibility in the cytosol (Fig. 8). In contrast, kinesin-1 does not appear to influence the Cdc42 pathway. These findings reveal how motor proteins finely regulate DC motility. Future studies will seek to determine whether kinesin-1 also locally contributes to the retraction of cell protrusions during DC migration in complex environments.

On the same lines, treatment with a combination of nocodazole with a Rock or formin inhibitor restored slow motility and the constriction passage time in Kif5b-deficient DCs but had no effect on WT DCs. These data confirm that microtubule depolymerization in a confined environment acts upstream of RhoA pathway activation by releasing key actomyosin regulators such as GEF-H1 (fig. S9). The factor called CLASP-1 is known to regulate microtubule dynamics by stabilizing their plus-end extremity and promoting their growth and has recently been identified as controlling the coordination of cortical contractility and nuclear translocation in cells migrating in a confined environment (*29*). Thus, our work and that of others emphasize the important role of microtubules in cell migration, with fine-tuning of the spatiotemporal coordination between actin polymerization and actomyosin-dependent contraction (*30*).

We were also intrigued to find that disruption of the microtubule network by nocodazole in WT DCs fully mimicked the phenotype observed for Kif5b-deficient DCs [i.e., the absence of slow motility phases, a longer constriction passage time (due to slower nuclear passage), and correction of these changes by treatment with a Rock or formin inhibitor]. This finding suggests that the regulation of kinesin-1’s motility along microtubules and the protein’s interaction with the actomyosin regulator GEF-H1 are involved in the spatiotemporal coupling between actin and microtubule cytoskeletons and thus promote efficient ameboid cell migration (Fig. 8 and fig. S9). This finding also highlights the complexity of the regulation of GEF-H1 release from microtubules, which can be induced by (i) microtubule disassembly, (ii) the microtubules’ acetylation status, and (iii) modulation of GEF-H1’s interaction with microtubule-dependent molecular motors (*13*, *16*, *18*). Further work should seek to clarify the importance of each mechanism involved in GEF-H1 release from microtubules, as a function of the migration mode and cell type.

We showed that human DCs can migrate in microchannels and investigated the molecular mechanisms involved in this process. We demonstrated that (as in mouse DCs) kinesin-1, microtubules, and the modulation of RhoA activity are required for efficient migration of human DCs in a confined environment. A better understanding of the molecular players regulating cell migration could (i) enable to develop new strategies for DC-based immunotherapy and (ii) circumvent one of the main limitations of this approach by increasing the ability of activated DCs to migrate into lymph nodes and thus to initiate a more efficient immune response (*31*, *32*).

In conclusion, the present study identified a previously unidentified role for the conventional microtubule-dependent motor protein kinesin-1 in the fine-scale regulation of the cross-talk between microtubules and actin during DC migration in humans and mice. By interacting with GEF-H1 (one of the key actomyosin regulators), kinesin-1 coordinates cytoskeletal interplay by modulating the RhoA activity required for efficient ameboid cell migration in a confined environment. Exploration of the molecular pathways controlling the spatiotemporal coordination between actin and microtubule cytoskeletons during cell migration could improve strategies for cell-based immunotherapy.

## MATERIALS AND METHODS

### Reagents and antibodies

Anti-CD11c [allophycocyanin (APC) (Sony Biosciences, 1186550) and BV711 (BioLegend)], anti-IA/IE [FITC (BD Biosciences), APC-Cy7 (BioLegend), and anti-CD8α BV450 (Sony Biosciences)], anti-CD11b [phycoerythrin (PE; BD Biosciences), APC-CY7 (Sony Bio-sciences), and FITC (Sony Biosciences, 1106025)], anti-F4/80 PE/Cy7 (Sony Biosciences), anti-CD205 PE/Cy7 (Sony Biosciences, 1291050), anti-GR1 APC (BD Biosciences), anti-CD19 [PerCP (BD Biosciences) and PerCP/Cy5.5 (Sony Biosciences)], anti-B220 BV650 (BioLegend), anti-CD86 BV650 (Sony Biosciences), anti-CD40 PE/Cy7 (Sony Biosciences, 1223110), anti-CD45 [V450 (BD Biosciences, 560501), FITC (BioLegend, 103108), anti–RhoA-GTP (NewEast Biosciences, #26904), anti-Cdc42 GTP mouse monoclonal antibody (NewEast Biosciences, #26905), mDia1 (BD Biosciences, clone 51/mDia1 610849), anti–GEF-H1 (Abcam, AB155785), anti-KIF5B (Proteintech), anti–glyceraldehyde-3-phosphate dehydrogenase (GAPDH; Sigma-Aldrich, G9295-200UL), anti-tubulin (Merck, T5168), phalloidin Alexa Fluor Plus 647 (Life Technologies, A30107), and phalloidin CruzFluor 488 Conjugate (Santa Cruz Biotechnology, sc-363791)], anti-CD86 Alexa Fluor 647 (AF647) (Sony Biosciences), anti–human leukocyte antigen–DR BV11 (BioLegend), anti-CD40 PE (BioLegend), anti-CD80 PE (Sony Biosciences), anti-CCR7 PE (BioLegend), anti-CD3/B220 PerCP (Sony Biosciences, 1116165), anti-CD64 APC (Sony Biosciences, 1296525), anti-Ly6C AF700 (BioLegend), Zombie Aqua Viability Kit (BioLegend), anti-XCR1 BV650 (BioLegend), anti-CD45.2 BV785 (BioLegend), and Live/Dead fixable Violet (Life Technologies, L34955) were used. AF488-conjugated dextran (10,000 kDa) was obtained from Life Technologies (D22910).

Calpeptin (Thermo Fisher Scientific, J60481.MA) was used at a concentration of 30 mg/ml. Tubacin (Sigma-Aldrich, SML0065) was used at a concentration of 0.5 μM. Y27632 (Sigma-Aldrich, Y0503) was used at a concentration of 30 μM. SMIFH2 (Sigma-Aldrich, S4826) was used at a concentration of 10 μM. Nocodazole (Sigma-Aldrich, M1404) was used at a concentration of 10 μM. MBQ167 (MedChemExpress, HY-112842) was used at a concentration of 100 nM. Cdc42 activator (Cytoskeleton, CS-CN02-A) was used at a concentration of 100 ng/ml.

### Animal statement

Housing and experiments complied with French and European Union regulations and guidelines. The protocol was approved by the local animal care and use committee (CEEA 34, Université Paris Cité, Paris, France, APAFIS#44108) under the number APAFIS#20-25050711568584.

### Mice

VAV-Cre transgenic mice (the Jackson Laboratory) were first crossed with *Kif5b*^+/−^ mice to generate *Kif5b*^+/−^;*VAV-Cre* animals. The latter were then bred with *Kif5b*^fl/fl^ mice to generate *Kif5b*^fl/−^;*VAV-Cre* (cKO^Kif5b^) mutant mice and control (WT) *Kif5b*^fl/+^ littermates. Mice were genotyped in a polymerase chain reaction (PCR) with the primers described by Cui *et al.* (*33*). All mice used in experiments were between 8 and 12 weeks old and were maintained under pathogen-free conditions.

### DC purification and BMDC and MDDC differentiation and culture

To purify DCs, spleens from wild-type or cKO^Kif5b^ mice were digested with Liberase (500 μg/ml; Roche Diagnostics) and recombinant deoxyribonuclease I (DNase I) (50 ng/ml; Roche Diagnostics) in phosphate-buffered saline (PBS). The DCs were preenriched from splenocytes, using a very-low-density gradient. Briefly, splenocytes were first resuspended in 4.2 ml of RPMI 1640 containing 5 mM EDTA, 5% fetal bovine serum (FBS), and 1 ml OptiPrep (Sigma-Aldrich) and then loaded between 3 ml of PBS containing 5 mM EDTA, 5% FBS, and 1 ml OptiPrep (bottom layer) and 1.8 ml of PBS containing 5 mM EDTA and 5% FBS (top layer). After 20 min of centrifugation at 1800 rpm at room temperature, the low-density fraction was collected at the interface between the top and middle layers. The cells were immunostained with the following antibodies (all diluted 1:200): anti-CD11c BV711, anti-IA/IE FITC, anti-CD8α BV450, anti-CD11b PE, anti-F4/80 PE/Cy7, anti-GR1 APC, anti-CD19 PerCP, and anti-B220 BV650. Live cells were gated on the basis of forward scatter and side scatter. Doublets were excluded, as were B cells (CD19^+^), macrophages (F4/80^+^) and granulocytes (Gr1^+^). The CD11c^+^ IA/IE^+^ population was selected, to separate CD8α^+^ DCs (*21*). The cells were sorted on a BD FACSAria II. For the CCR7 staining, several adjustments were made to the antibody panel. The following antibodies were used: anti-CCR7 PE, anti-CD11b FITC, anti-CD3/B220 PerCP, anti-F4/80 PE-Cy7, anti-CD64 APC, anti-Ly6C AF700, anti-MHCII APC-Cy7, Zombie Aqua, anti-XCR1 BV650, anti-CD11c BV711, and anti-CD45.2 BV785.

Cells were acquired on the NovoCyte Flow Cytometer. Live cells were identified using Zombie Aqua viability dye, and doublets were excluded. Macrophages (CD64^+^/F4/80^+^) and lymphocytes (B220^+^/CD3^+^) were also excluded. The subsequent gating strategy focused on selecting CD11c^+^/MHCII^+^ cells to isolate cDCs. Among these, XCR1^+^ cells were further gated to obtain the cDC1 population.

For the generation of BMDCs, bone marrow was isolated from femurs of 8- to 12-week-old mice. Cells were then cultured in medium supplemented with granulocyte-macrophage colony-stimulating factor (GM-CSF), 15% heat-inactivated FBS, 1% nonessential amino acids, 1 mM sodium pyruvate, penicillin (100 U/ml), and streptomycin (100 U/ml). After 8 to 10 days of culture, the BMDCs were used in experiments.

To generate MDDCs, peripheral blood mononuclear cells (PBMCs) were isolated from buffy coats obtained from healthy human blood donors (Etablissement Français du Sang, authorization 2022-2026-046 CCPSL IMAGINE) using isolation with Ficoll-Paque separation medium (GE Healthcare). Monocytes were obtained after labeling the PBMCs with anti-human CD14 magnetic beads (Miltenyi) and cultured in RPMI (Gibco), 10% FBS (heat-inactivated, Sigma-Aldrich), 10 mM Hepes, 55 μM β-mercaptoethanol, 6 mM l-glutamine, and penicillin/streptomycin/gentamycin (Gibco), human recombinant GM-CSF (10 ng/ml), and human recombinant interleukin-4 (IL-4) (50 ng/ml; Miltenyi). After 24 and 72 hours of culture, fresh medium (40% of the total volume) with cytokines was added. Differentiation was completed on day 4 after monocyte purification.

### RNA sequencing

Using 1.5-ml ribonuclease-free Eppendorf tubes, cDC1s were sorted from the spleen of three control mice and five cKO^Kif5b^ mice in 300 μl of lysis buffer from the RNeasy Plus Micro Kit (QIAGEN). After RNA extraction, the RNA concentration was measured in a fluorometric Qubit RNA assay (Life Technologies). The RNA integrity number (RIN) was determined on the Agilent 2100 Bioanalyzer (Agilent Technologies) with the Pico Chip Kit, as per the manufacturer’s instructions. To construct the libraries, 100 ng of high-quality total RNA sample (RIN > 6.5) was processed using NEBNext polyadenylate library prep kit, according to the manufacturer’s instructions. Briefly, total RNA molecules were reverse transcribed using oligo(dT) primers and template switching oligos. The cDNA was then amplified with 3′ and 5′ adapters and quality controlled. After fragmentation, the amplified cDNA was end repaired and ligated with NEBNext adapters. PCR enrichment with a single barcode gave the final library. Libraries were then quantified with a Qubit HS DNA assay (Life Technologies), and the library profiles were assessed using the DNA High Sensitivity LabChip Kit on the Agilent 2100 Bioanalyzer. Libraries were sequenced on an Illumina NextSeq 500 instrument, using 75–base length read V2 chemistry in a paired-end mode. After sequencing, AOZAN software (ENS, Paris) was used to demultiplex and control the quality of the raw data (based on FastQC modules, version 0.11.5).

### RNA sequencing analysis

FASTQ files were mapped to the Ensembl mouse genome assembly GRCm39/mm39 reference using DRagen and counted via the featureCounts routine in the Subread package (https://subread.sourceforge.net/). Read counts were normalized and groups were compared by applying three independent but complementary statistical methods: Deseq2, LimmaVoom, and edgeR. Flags were calculated from counts normalized against the mean coverage. All normalized counts of <20 were considered to be background (flag 0) and those of ≥20 were considered to be signal (flag = 1). The P50 lists used for the statistical analysis regrouped together the genes with flag = 1 for at least half of the compared samples. The results of the three methods were filtered with *P* ≤ 0.05 and a fold change of ≥1.2, compared, and grouped in a Venn diagram. Gene lists common to all three methods were uploaded to Ingenuity Pathway Analysis (QIAGEN). Hierarchical clustering was assessed with the Spearman correlation similarity measure and Ward’s linkage algorithm. Heatmaps were drawn with the NMF package and the Viridis package’s magma color palette in R software.

For the enrichment analysis, lists were generated by selecting up-regulated versus down-regulated and DEGs, relative to reference populations. A custom multiple gene lists analysis was performed for DEGs (analyzed as *Mus musculus* species) using Metascape (https://metascape.org). DEGs with a minimum 20 overlap of 3, a *P* value cutoff of 0.01, and a minimal enrichment of 1.5 were selected for the pathway and process enrichment analysis. The input genes lists included were GO Molecular Functions (Functional Set) and GO BPs. The output lists of terms were filtered to select pathways with adjusted *P* values (*q* values) of <0.01. The selection criteria for the plotted 10 pathways are given in the figure legends.

### In vivo competitive homing experiments

BMDCs were concentrated to 10^6^ cells/ml in PBS and treated with LPS (10 g/ml for 10 min), before labeling with either carboxyfluorescein diacetate succinimidyl ester (CFSE) (Invitrogen) or a Far Violet Cell Staining Kit (Invitrogen). CFSE and Far Violet were used to distinguish between WT and Kif5b-deficient labeled DCs upon coinjection. Cells were washed twice in PBS–0.5% bovine serum albumin (BSA), resuspended in PBS–0.1% BSA at 10 × 10^6^ cells/ml, incubated with CFSE (5 μM) or Far Violet (5 μM) for 30 min at 37°C, and extensively washed with PBS–0.0.5% BSA. Cells were resuspended in PBS to 10^8^ cells/ml and mixed 1:1 with the other DC type. Next, 20 μl of the cell mix was injected into the footpad of recipient C57/BL6 mice. Twenty microliters was kept and analyzed with flow cytometry on the day of the injection to confirm the 1:1 ratio. Twenty-four hours later, popliteal LNs and footpads were collected, treated with collagenase D (1 mg/ml; Roche Diagnostics) and DNase I (Roche Diagnostics) for 45 min at 37°C, mashed, stained, and analyzed with flow cytometry. The percentage of migrated DCs corresponded to the ratio between fluorescent DCs and total LN cells. The single-cell suspension obtained from popliteal LNs was stained with anti-CD11c and anti-CD205 antibodies.

Additional in vivo competitive homing experiments were performed using Kif5b-deficient BMDCs only. Cells were treated (or untreated) with 30 mM Y27632 (Sigma-Aldrich) for 2 hours before labeling with CFSE or a Far Violet. CFSE and Far Violet were used to distinguish between the treated and untreated Kif5b-deficient DCs upon coinjection.

### FITC ear painting

cKO^Kif5b^ and C57BL/6JRj mice aged between 6 and 8 weeks were used under anesthesia. One ear per mouse was painted with 25 μl of 1% FITC (Sigma-Aldrich) in 1:1 acetone/dibutyl phthalate. The solution was dropped onto the ear, which was then rubbed to ensure that all the solution penetrated into the ear tissue. After 24 hours, mice were euthanized, and dLNs were collected. Tissues were mechanically disrupted, digested with collagenase D (1 mg/ml; Merck-Roche) and DNase I (50 μg/ml; Merck-Roche) for 45 min, and filtered through a 70-μm cell strainer to give a single-cell suspension. Cells were then stained for CD11c, CD45, and CD40 and analyzed with flow cytometry.

### Migration speed measurement in microchannels

Microchannels were prepared as described previously (*25*, *34*). Polydimethylsiloxane (PDMS; GE Silicones) was used to prepared 4 or 8 μm–by–5 μm microchannels or microchannels with constrictions, using a custom-made mold. The PDMS chamber and a 35-mm glass-bottom dish (World Precision Instruments, FD35-100) were plasma activated before being bound to each other. The bonding was left to strengthen in a 70°C oven for 1 hour. The microchannels were then plasma cleaned, coated with fibronectin (10 μg/ml; Sigma-Aldrich) or with PEG at room temperature for 1 hour, and then washed three times with PBS. The nuclei of BMDCs or MDDCs were stained for 30 min at 37°C with Hoechst reagent, and the cells were then washed and seeded at of 1 × 10^5^ BMDCs or MDDCs in complete medium containing cytokines [GM-CSF (50 ng/ml) for BMDCs and GM-CSF (10 ng/ml) and IL-4 (50 ng/ml) for MDDCs] in the wells of the devices. We imaged migrating BMDCs or MDDCs for 16 hours with a Zeiss Axio Observer Z1 equipped with Yokogawa spinning disk technology, a Hamamatsu digital camera C11440 (Carl Zeiss), and a 10× numerical aperture (NA) of 0.45 objective. One image was acquired per 2 min of transmission phase. We generated kymographs of the migrating cells by subtracting the mean projection of the whole movie to each frame and generating clear objects in dark background using a custom program, as we described previously (*35*). For the chemotactic response assay, two wells were made on either side of the microchannels in the PDMS device. Mouse recombinant CCL19 (PeproTech, #250-27B-20UG) was added to the well opposite the cell seeding site immediately before image acquisition.

### Drugs treatment

Dimethyl sulfoxide was used as a control for all drugs other than Y27632, which was dissolved in water. When drugs were used in microchannel experiments, the chamber was incubated with drug-containing medium before cell loading. Cells were resuspended in drug-containing medium 1 hour before being deposited in the chamber entry pores.

### Macropinocytosis in migrating BMDCs

BMDCs were stained with 4′,6-diamidino-2-phenylindole before loading into 5 μm–by–8 μm microchannels prefilled with 10-kDa AF488-conjugated dextran (120 μg/ml; Life Technologies) for 16 hours. The number and surface area of dextran-internalized macropinosomes were quantified by drawing regions of interest using the QuPath software (v0.5.1).

### Immunofluorescence

Actin, mDia1, GEF-H1, and tubulin distributions were analyzed on BMDCs loaded on 5 μm–by–8 μm microchannels for 16 hours. The cells in the channels were then fixed with 3% paraformaldehyde for 20 min and washed in PBS. The PDMS structure on top of the channels was then removed carefully. The cells were permeabilized with 0.25% saponin in PBS for 10 min; stained with rhodamine-conjugated phalloidin, mDia1, tubulin, or GEF-H1; and imaged using a Zeiss Axio Observer Z1 equipped with Yokogawa spinning disk technology and a 63× NA of 1.46 objective.

### Density map

Density maps were generated using ImageJ software (version 1.43). Briefly, images were cropped to contain single cells and resized to the mean cell size. After background subtraction, the intensities were normalized, and density maps were generated by projecting the mean signal of every individual cell and applying the physics look-up table to the image. Intensities were measured in rectangles covering the front 20% of the cell and the rear 20% of the cell.

### Measurement of GEF-H1 fluorescence intensity associated with microtubules

Image analysis was performed using QuPath software (v0.5.1). A pixel classifier was trained on the α-tubulin channel to generate a binary mask segmenting the microtubule network. This mask was applied to the GEF-H1 fluorescence channel to selectively quantify microtubule-associated GEF-H1 signal. Mean fluorescence intensity (MFI) of GEF-H1 within the tubulin-defined mask was then calculated for both WT and cKO^Kif5b^ DCs.

### Lentivirus production and transduction

The pLK0.1-puro plasmids carrying a puromycin resistance gene and shRNA sequences specific for mouse ARHGEF2 (GEF-H1) [TRCN0000109985 (shRNA^85^), TRCN0000109986 (shRNA^86^), and TRCN0000109987 (shRNA^87^)] or a nontargeting shRNA sequence [SHC016-1EA (shRNA^sc^)] were purchased from Sigma-Aldrich. The pLK0.1 plasmids were cotransfected with the packaging plasmid pCMV-VSV-G and the envelope plasmid psPAX2 into human embryonic kidney (HEK) 293FT cells (Life Technologies) using Jetprime (Sartorius) transfection. Five hours posttransfection, the buffer was exchanged for complete Dulbecco’s modified Eagle’s medium (DMEM), and the virus-containing supernatant was collected 24, 36, and 72 hours posttransfection. The supernatants were filtered through 0.45-μm filters (Merck Millipore) and concentrated by ultracentrifugation for 2 hours at 70,000*g*.

Red cell–depleted bone marrow cells on day 3 of culture in the presence of GM-CSF cells were put into contact with lentiviral supernatants at a multiplicity of infection of 10, supplemented with polybrene (8 μg/ml), and spinoculated for 100 min at 2200 rpm at 37°C. Subsequently, supernatants were washed off and replaced with BMDC culture medium. Nontransduced cells were depleted by the addition of puromycin (5 μg/ml) on day 5. Transduced cells were used on days 7 or 8 of culture. The efficiency of knockdown was confirmed by immunoblotting.

To produce virus and virus-like particles (VLPs) for KIF5B knockdown in MDDCs, HEK293FT cells were transfected in 10-cm plates with 1.2 ml of DMEM (Life Technologies) containing 18 μg of DNA and 48 μl of TransIT-293 (Mirus Bio) per plate in a 10-ml final volume, according to the transfection reagent manufacturer’s protocol. For the production of VSV-G–pseudotyped SIVmac VLPs containing Vpx, 18 μl of DNA consisted of 2.4 μg of pTRIP CMV-VSV-G and 15.6 μg of pSIV3^+^. For the production of lentiviral particles for stable expression of shRNAs, 18 μg of DNA consisted of 9.6 μg of a pLKO.1 vector coding for shRNAs [Sigma Merck mission KIF5B shRNA 1 (TRCN0000113871), shRNA 2 (TRCN0000113872), shRNA 3 (TRCN0000113873), shRNA 4 (TRCN0000113874), KIF5B shRNA 5 (TRCN0000113875), or scrambled shRNA], 6 μg of pCMV-ΔR8.91, and 2.4 μg of pTRIP CMV-VSV-G. Production was scaled up to 15-cm plates when greater volumes of Vpx-containing VLPs were needed, using the same ratios. Twenty-four hours after transfection, the media were changed; a further 24 hours later, viral supernatants were collected and filtered twice (0.45 μm; Merck Millipore).

To knock down KIF5B through stable shRNA expression in MDDCs, human monocytes were plated at 1 × 10^6^ cells/ml in 5 ml of medium with cytokines (GM-CSF and IL-4; see the “DC purification and BMDC and MDDC differentiation and culture” section for more details) and polybrene (8 μg/ml; Merck Millipore). The cells were transduced with 2.5 ml of supernatants containing SIVmac239 VLPs for Vpx delivery [see (*36*), (*37*), and the brief description below] and 4 ml of viral supernatant containing shRNA-encoding lentiviruses. Fresh media and cytokines were added to cells (40% of the total volume) 1 and 3 days after CD14^+^ cell isolation. On day 5, differentiated MDDCs were collected, resuspended in fresh media with cytokines, and used in migration experiments. Maturation marker expression was assessed with flow cytometry.

### Immunoprecipitation and immunoblotting

For the immunoprecipitation assay, BMDCs were lysed in lysis buffer [50 mM Hepes (pH 7.4), 150 mM NaCl, 1 mM MgCl_2_, 1% Triton X-100, and 10% glycerol] supplemented with an EDTA-free protease inhibitor cocktail tablet and phosphatase inhibitors (Sigma-Aldrich). Proteins were immunoprecipitated with an anti-GEF-H1 antibody (Abcam), polyclonal rabbit KIF5B antibody (Proteintech), or an isotype control (normal rabbit immunoglobulin G polyclonal antibody, Merck). Anti–GEF-H1 and anti-KIF5B antibodies were used to develop the Western blots.

BMDCs were lysed in lysis buffer [50 mM tris-HCl (pH 7.6), 150 mM NaCl, 5 mM EDTA, and 0.5% Triton X-100] supplemented with an EDTA-free protease inhibitor cocktail tablet (Roche Diagnostics). Proteins were separated by SDS–polyacrylamide gel electrophoresis and transferred to nitrocellulose membranes. Membranes were incubated with anti–GEF-H1 or anti-GAPDH primary antibodies. Immunoreactive bands were visualized with enhanced chemiluminescence detection reagents (Thermo Fisher Scientific), using an imaging system for chemiluminescence (Fusion FX, Vilber). The band intensity in the Western blots was quantified using ImageLab software (Bio-Rad).

### Statistical analysis

All statistical analyses were performed with GraphPad Prism software (version 9; GraphPad Software, La Jolla, CA). Unpaired Student’s *t* test, one-way analysis of variance (ANOVA) with multiple comparisons (Kruskal-Wallis), two-way ANOVA using Sidak’s correction, or Wilcoxon matched-pairs test was performed to determine the statistical significance of differences between groups. The threshold for statistical significance was set to *P* < 0.05. *P < 0.05, **P < 0.01, ****P < 0.001, and ****P < 0.0001. Data represent the means ± SD. In the box plot, the box depicts the interquartile range, the center line corresponds to the median, and the whiskers depict the 10th to 90th percentiles.
