# Supplementary material for: Kinesin-1 coordinates cross-talk between microtubule and actin cytoskeletons during dendritic cell migration
Source: Sci Adv. 2025 Oct 17;11(42):eadx7672. doi: 10.1126/sciadv.adx7672 (PMC12533555; doi:10.1126/sciadv.adx7672)
Supplement: Supplementary file 1 — Figs. S1 to S9 Legends for movies S1 to S21 [file sciadv.adx7672_sm.pdf]

Science Advances
AAAS

Supplementary Materials for

Kinesin-1 coordinates cross-talk between microtubule and actin cytoskeletons during dendritic cell migration

Pierre Duquesne *et al.*

Corresponding author: Gaël Ménasché, gael.menasche@inserm.fr

Sci. Adv. **11**, eadx7672 (2025)
DOI: 10.1126/sciadv.adx7672

The PDF file includes:

Figs. S1 to S9
Legends for movies S1 to S21

Other Supplementary Material for this manuscript includes the following:

Movies S1 to S21

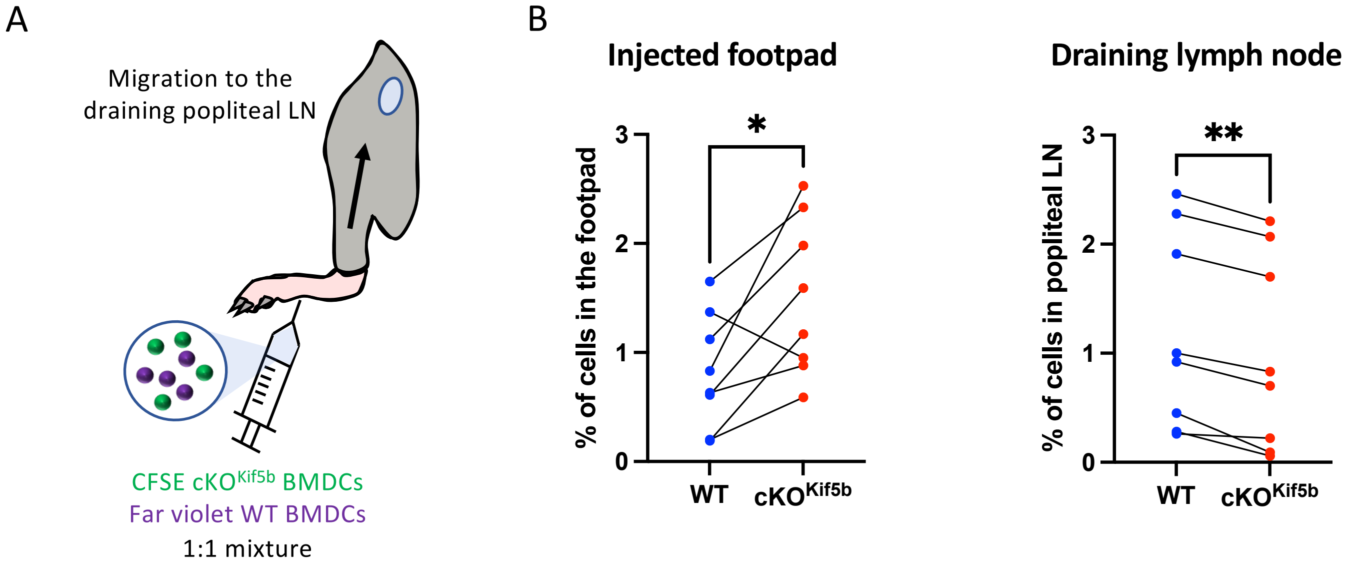

Figure S1: *In vivo* competitive homing experiment. A) Graphical summary of the competitive homing assays. WT BMDCs were stained with Far Violet, and cKO^{Kif5b} BMDCs were stained with CFSE. Cells were then co-injected into the footpad of WT recipients. 24h after the injection, popliteal LNs and footpads were collected. FACS was performed to assess the proportion of cells having reached the draining LN or having stayed in the footpad. B) Quantification of recovered WT and cKO^{Kif5b} BMDCs in the injected footpad and draining popliteal lymph node (WT recipient mice n=8). Statistical significance was determined using Wilcoxon's matched pairs test (**p<0.01; *p<0.05).

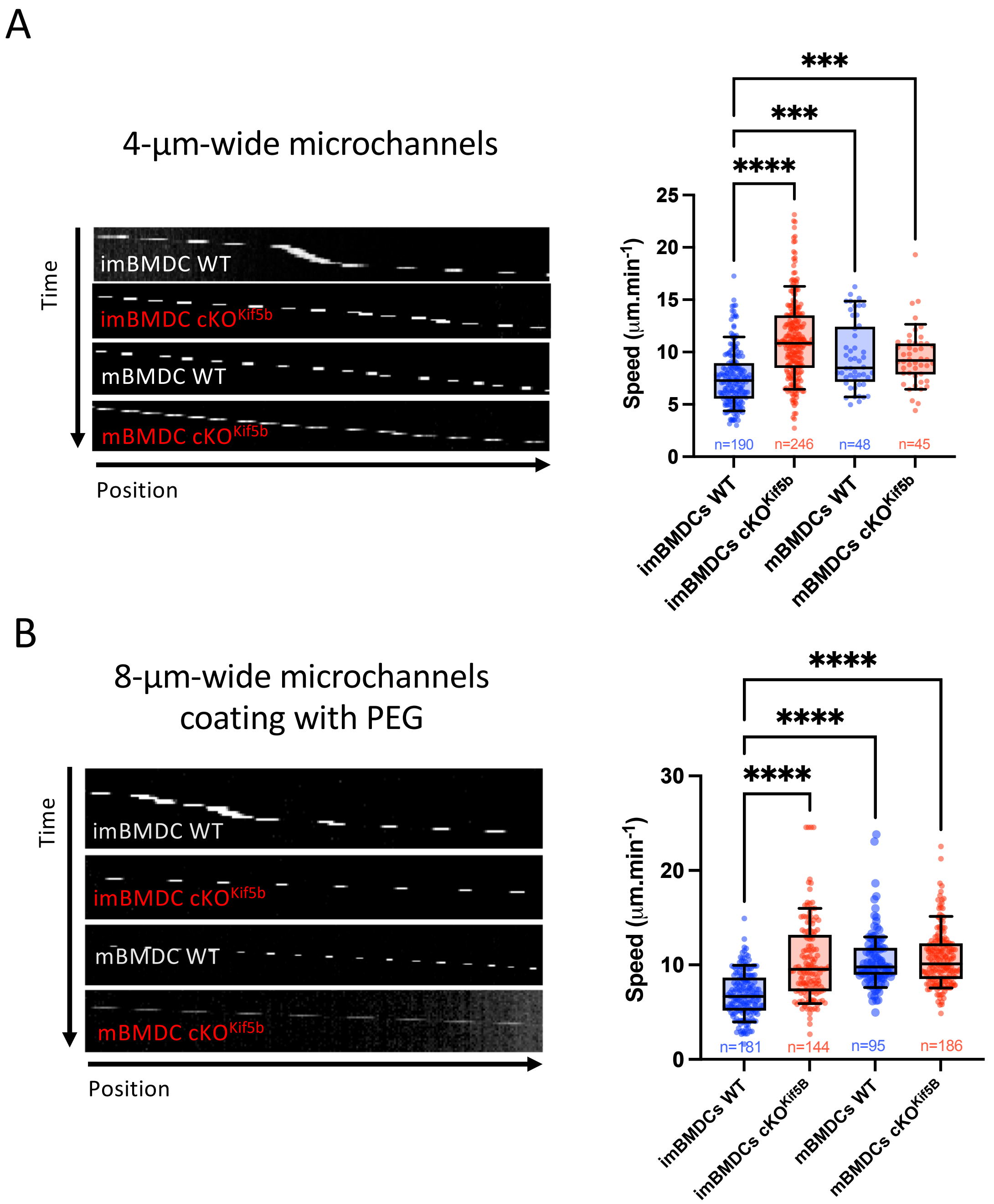

Figure S2: Kinesin-1 is required for slow migration by immature BMDCs in a confined environment. A) Left panel: Representative kymographs are shown for immature (im) and mature (m) WT and cKO^{Kif5b} BMDCs migrating within 4-µm-wide microchannels. Right panel: Quantification of the mean speed of immature and mature WT and cKO^{Kif5b} BMDCs. The number of cells (n) is indicated for each condition, pooled from N=3 independent experiments. Statistical significance was determined in a non-parametric one-way analysis of variance (ANOVA) with multiple comparisons (Kruskal-Wallis) (****p<0.0001; ***p<0.001). B) Left panel: Representative kymographs are shown for immature (im) and mature (m) WT and cKO^{Kif5b} BMDCs migrating within 8-µm-wide microchannels coating with PEG. Right panel: Quantification of the mean speed of immature and mature WT and cKO^{Kif5b} BMDCs. The number of cells (n) is indicated for each condition, pooled from N=3 independent experiments. Statistical significance was determined in a non-parametric one-way ANOVA with multiple comparisons (Kruskal-Wallis) (****p<0.0001).

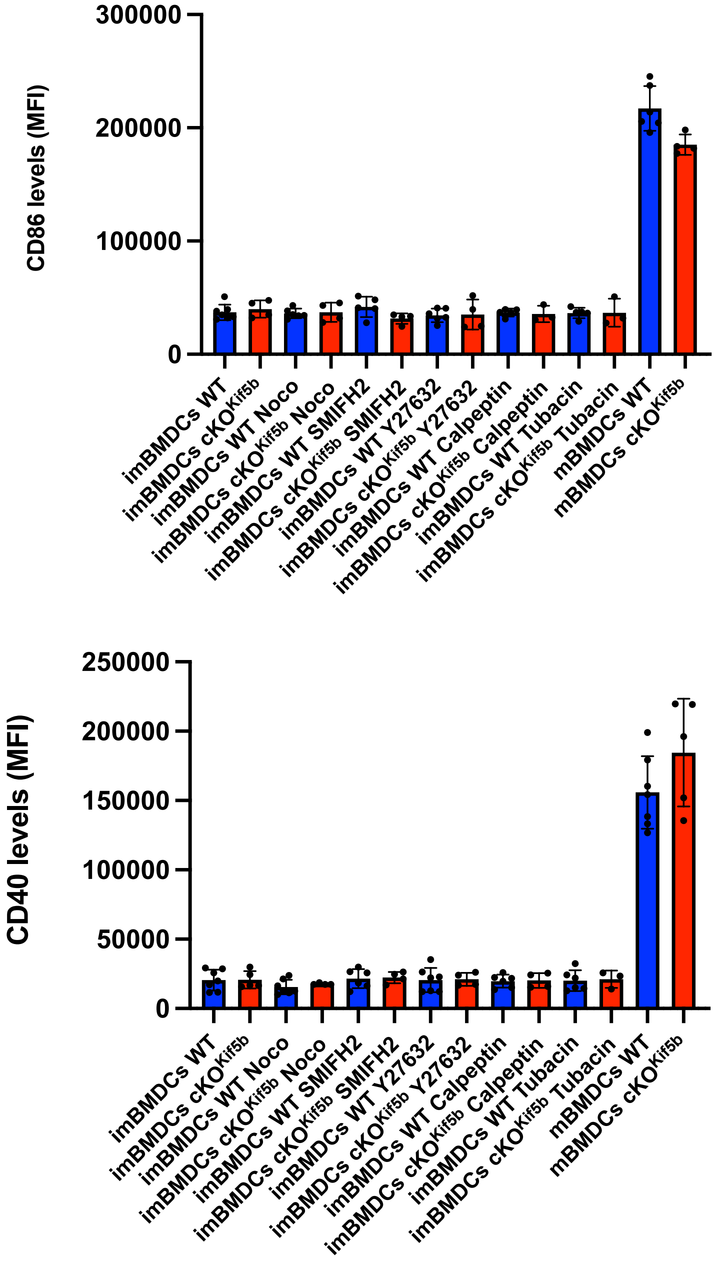

Figure S3: WT or cKO^{Kif5b} BMDCs treated with different drugs did not modulate their activation status. Flow cytometry quantification of CD86 and CD40 expression of WT or cKO^{Kif5b} immature (im) BMMCs treated (or not) with nocodazole (Noco), SMIFH2, Y27632, calpeptin, or tubacin. WT or cKO^{Kif5b} mature (m) BMDCs were activated for four hours with 1 µg/mL lipopolysaccharide (LPS). The data are pooled from N=6 independent experiments.

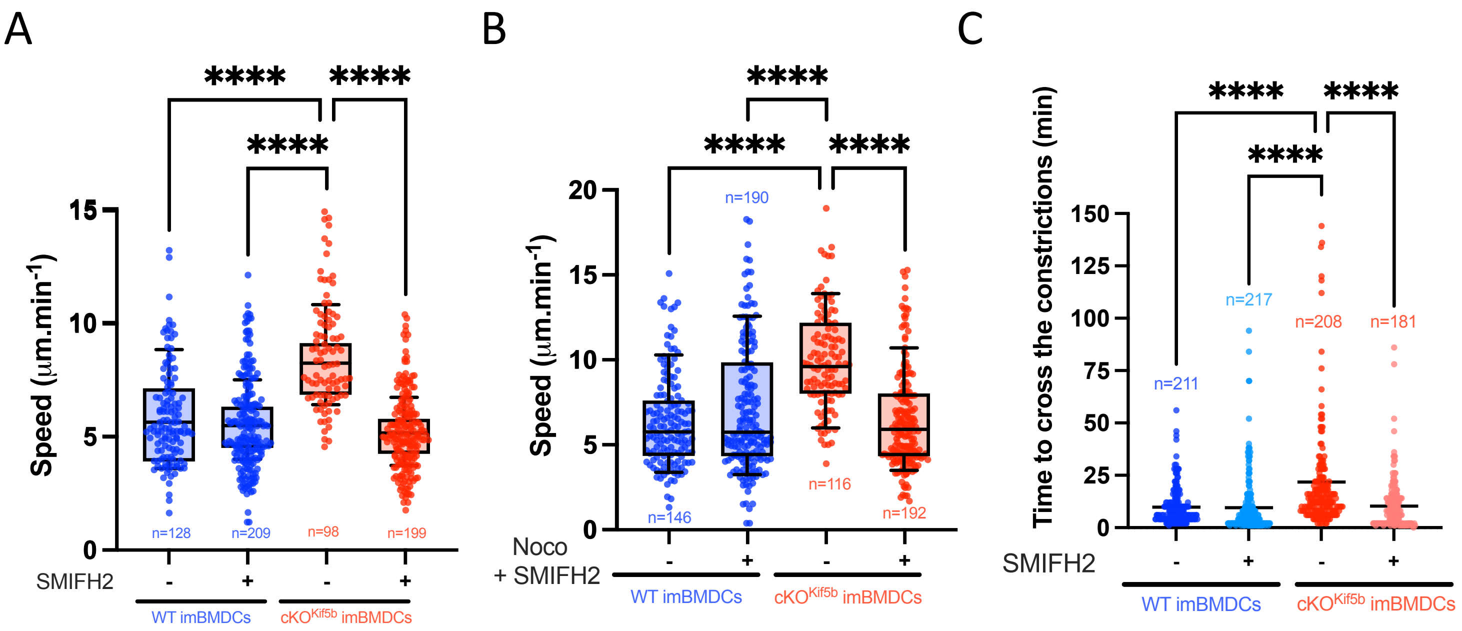

Figure S4: The formin inhibitor (SMIFH2) treatment restored the confined migration of cKO^{Kif5b}-deficient BMDCs. A-B) Quantification of the mean speed of immature WT and cKO^{Kif5b} BMDCs treated (or not) with SMIFH2 (A) or nocodazole (Noco) and SMIFH2 (B) with in 8 µm diameter microchannels. The number of cells (n) is indicated for each condition, pooled from N=2 or 3 independent experiments. Statistical significance was determined in a non-parametric one-way analysis of variance (ANOVA) with multiple comparisons (Kruskal-Wallis) (*****p*<0.0001). C) Quantification of the time needed to pass through 1.5 µm constrictions for WT and cKO^{Kif5b} imBMDCs treated (or not) with SMIFH2. The number of cells (n) is indicated for each condition, pooled from N=2 independent experiments. Statistical significance was determined in a non-parametric one-way ANOVA with multiple comparisons (Kruskal-Wallis) (*****p*<0.0001)

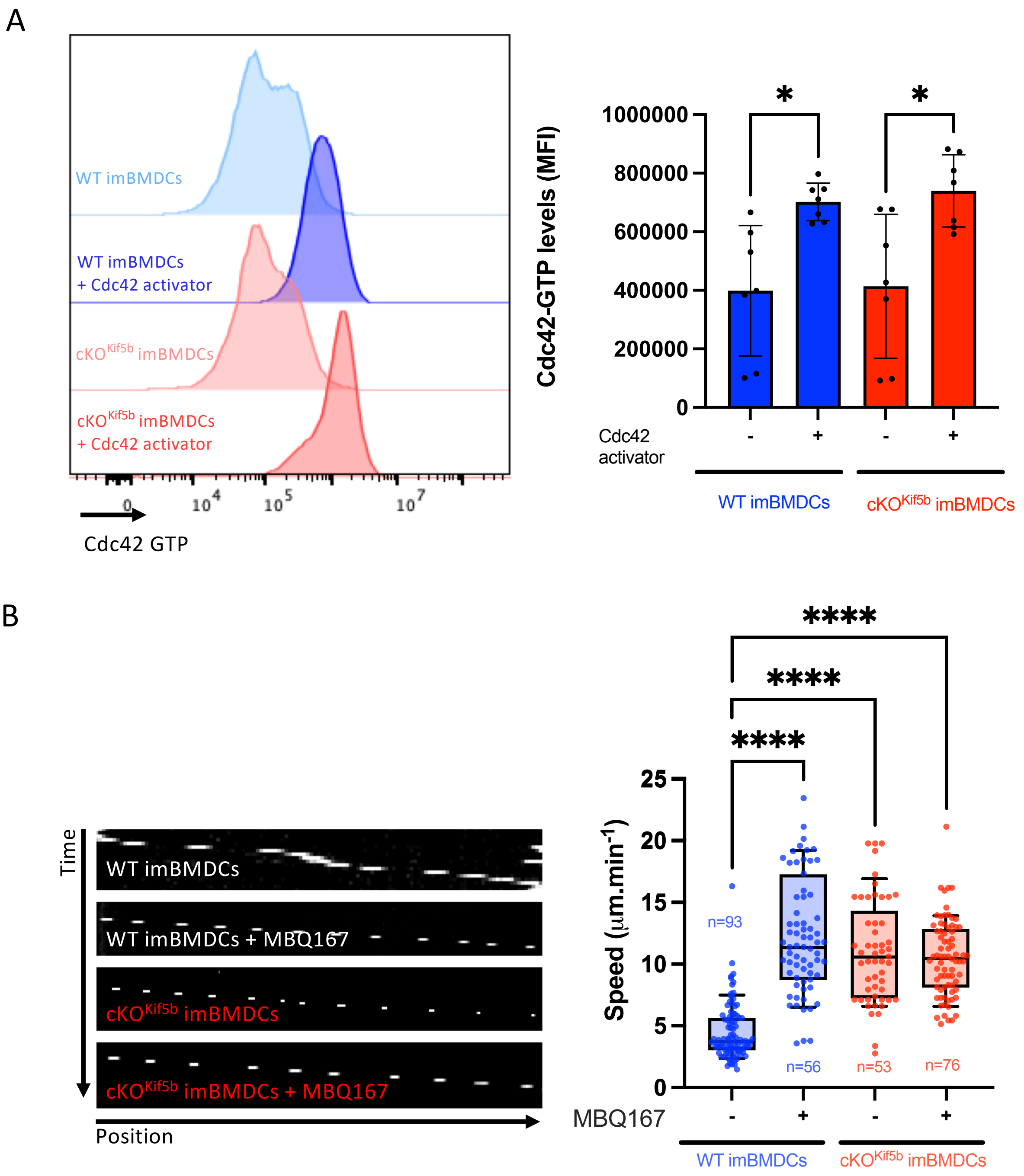

Figure S5: **Kinesin-1 deficiency does not alter Cdc42-mediated signaling.** A) Left panel: Flow cytometry quantification of Cdc42-GTP in WT and cKO^{Kif5b} imBMDCs treated (or not) with Cdc42 activator. Right panel: Cdc42-GTP levels from WT and cKO^{Kif5b} imBMDCs is shown (N=2 independent experiments). Statistical significance was determined in a non-parametric one-way analysis of variance (ANOVA) with multiple comparisons (Kruskal-Wallis) (**p<0.01). B) Left panel: Representative kymographs are shown for immature (im) WT and cKO^{Kif5b} BMDCs treated (or not) with MBQ167 with in 8 µm diameter microchannels. Right panel: Quantification of the mean speed of immature WT and cKO^{Kif5b} BMDCs treated (or not) with MBQ167. The number of cells (n) is indicated for each condition, pooled from N=2 independent experiments. Statistical significance was determined in a non-parametric one-way ANOVA with multiple comparisons (Kruskal-Wallis) (****p<0.0001).

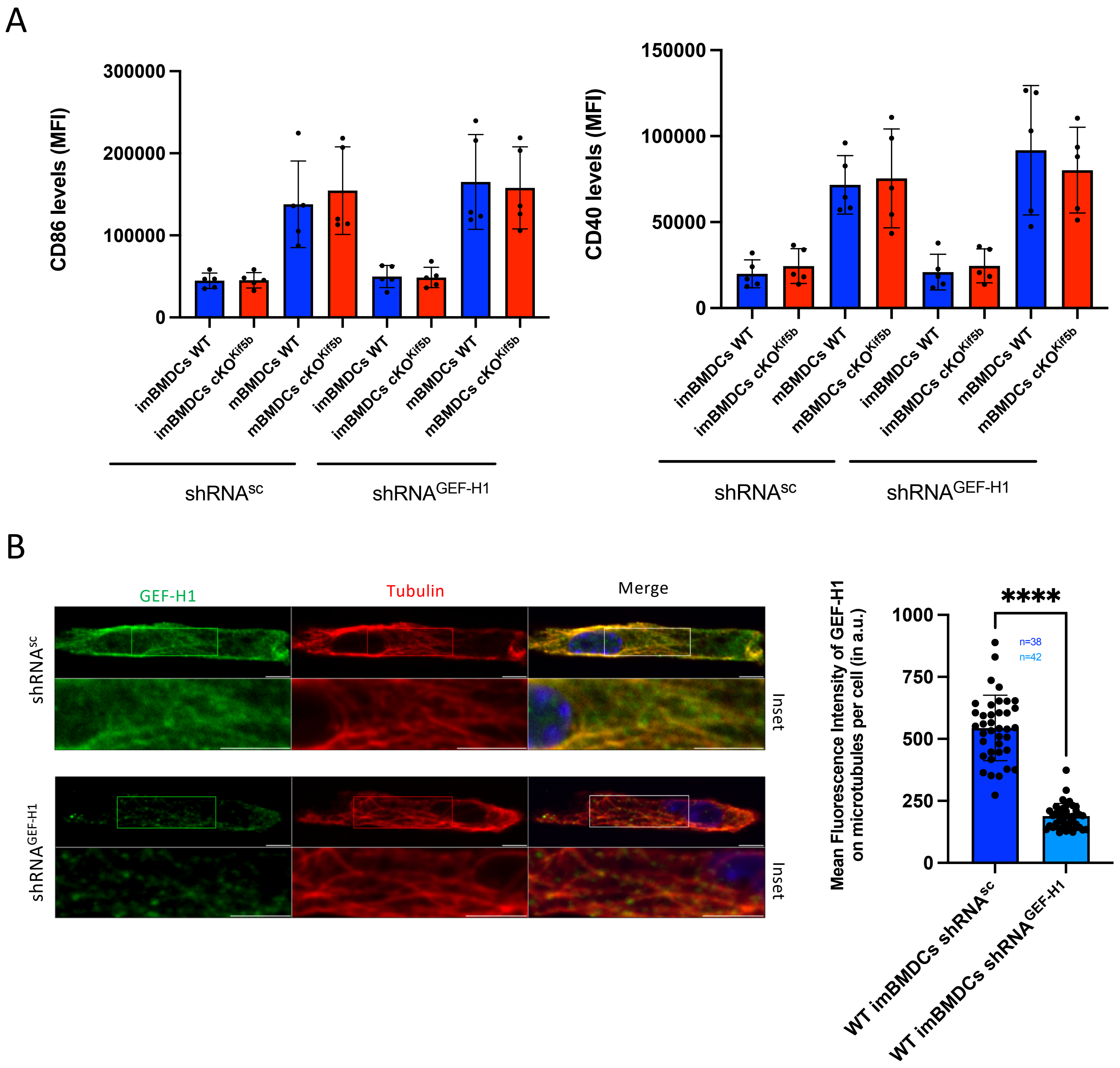

Figure S6: Effect of GEF-H1 knockdown on BMDC activation and staining. A) Infection of WT or cKO^{Kif5b} BMDCs with shRNA^{GEF-H1} or shRNA^{sc} did not modulate their activation status. Flow cytometry quantification of CD86 and CD40 expression of WT or cKO^{Kif5b} immature (im) BMMCs infected with shRNA^{GEF-H1} or shRNA^{sc}. Infected WT or cKO^{Kif5b} mature (m) BMDCs were activated for four hours with 1 µg/mL lipopolysaccharide (LPS). The data are pooled from N=5 independent experiments. B) Left panel: GEF-H1 and tubulin staining in WT or cKO^{Kif5b} immature (im) BMMCs, infected with shRNA^{GEF-H1} or shRNA^{sc}, during migration through 8 µm microchannels (n>38 cells/condition, from N=3 independent experiments). Right panel: Quantification of the mean fluorescence intensity of GEF-H1 on microtubules per cell is shown. Statistical significance was determined in an unpaired *t*-test (*****p*<0.0001).

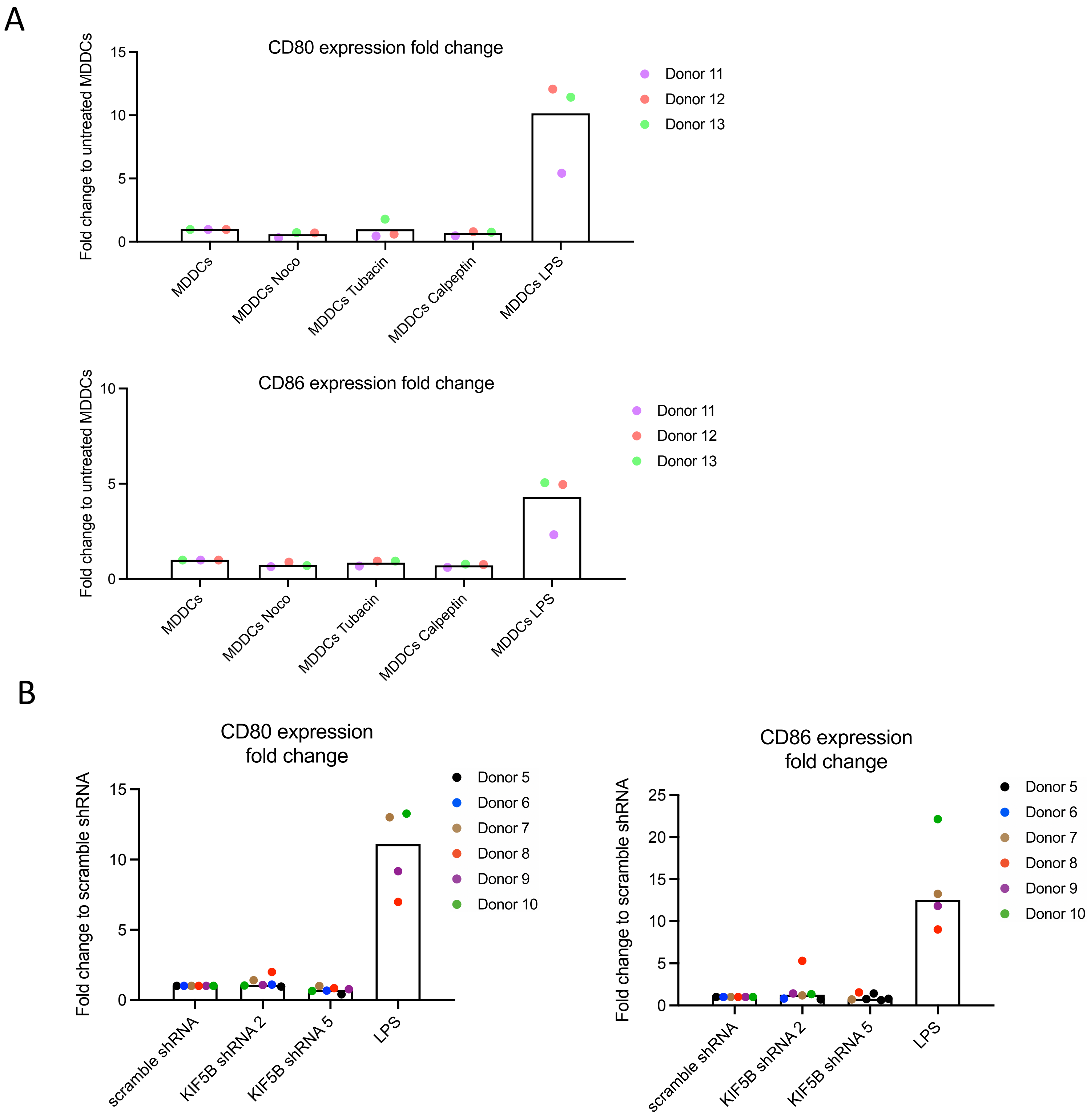

Figure S7: Treatment with various drugs or infection with shRNA^{KIF5B} or shRNA^{sc} does not modulate the activation status of human MDDCs. A) Flow cytometry quantification of CD80 and CD86 expression by MDDCs from various donors treated (or not) with nocodazole (Noco), calpeptin, tubacin, or activated with lipopolysaccharide (LPS). The data show the fold change in CD80 or CD86 expression, relative to untreated MDDCs. B) Flow cytometry quantification of CD80 and CD86 expression of MDDCs from various donors infected with shRNA n°2 or n°5 targeting KIF5B or control shRNA (shRNA^{sc}) or activated with LPS. The data show the fold change in CD86 or CD80 expression, relative to shRNA^{sc}.

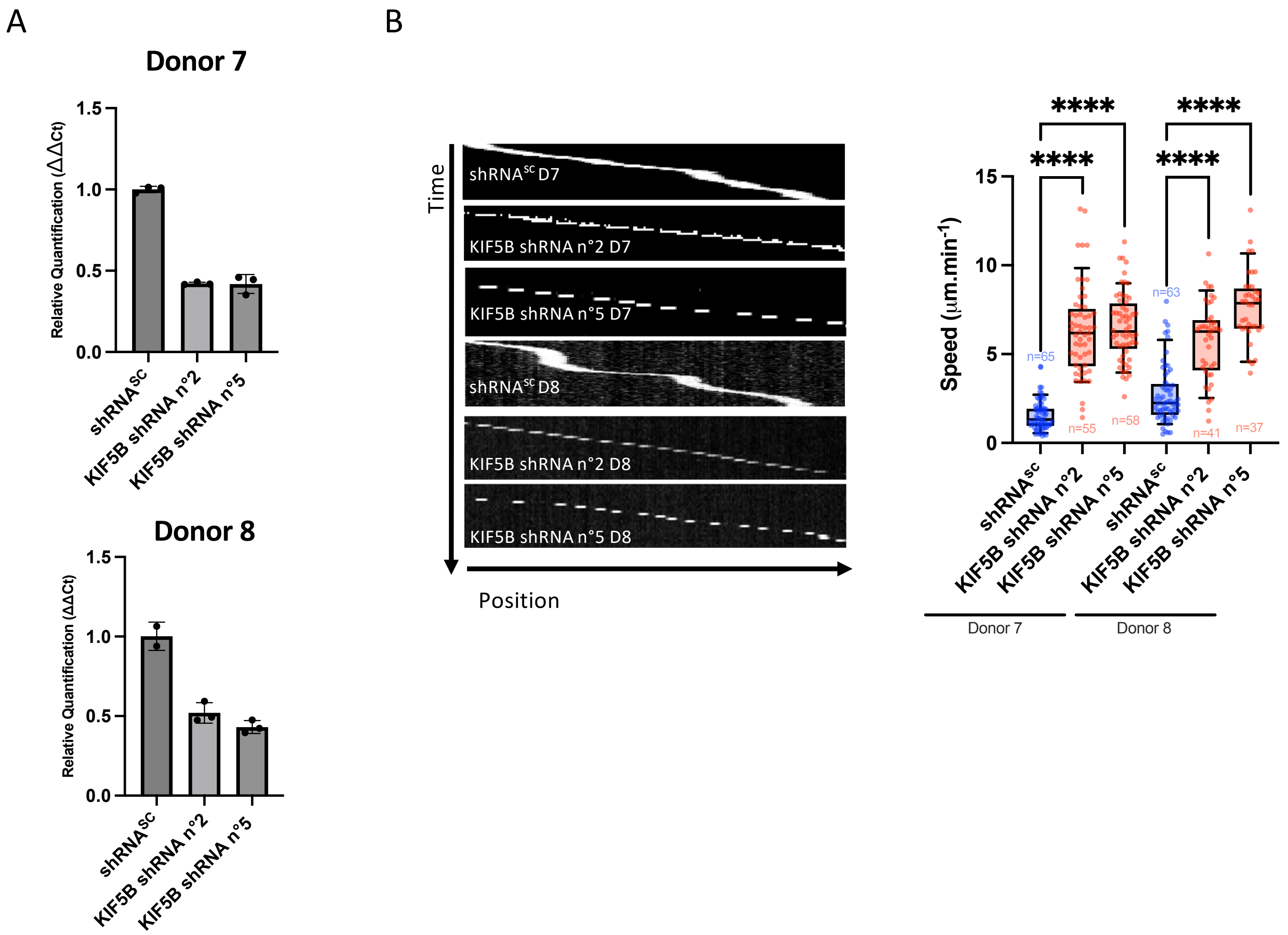

Figure S8: Kinesin-1 is required for the efficient migration of MDDCs in a confined environment. A) MDDCs from two donors (D7 and D8) were infected with viral particles expressing shRNA n°2 or n°5 (targeting KIF5B) or control shRNA (shRNA^{sc}). KIF5B transcript levels were determined by a real-time PCR assay. B) Left panel: Representative kymographs are shown for MDDCs from two donors (D7 and D8) infected with one of two shRNAs targeting KIF5B or with control shRNA (shRNA^{sc}) and migrating in 8 µm microchannels. Right panel: Quantification of the mean speed of the infected MDDCs. The number of cells (n) is indicated for each condition. Statistical significance was determined in a non-parametric one-way analysis of variance (ANOVA) with multiple comparisons (Kruskal-Wallis) (****p<0.0001).

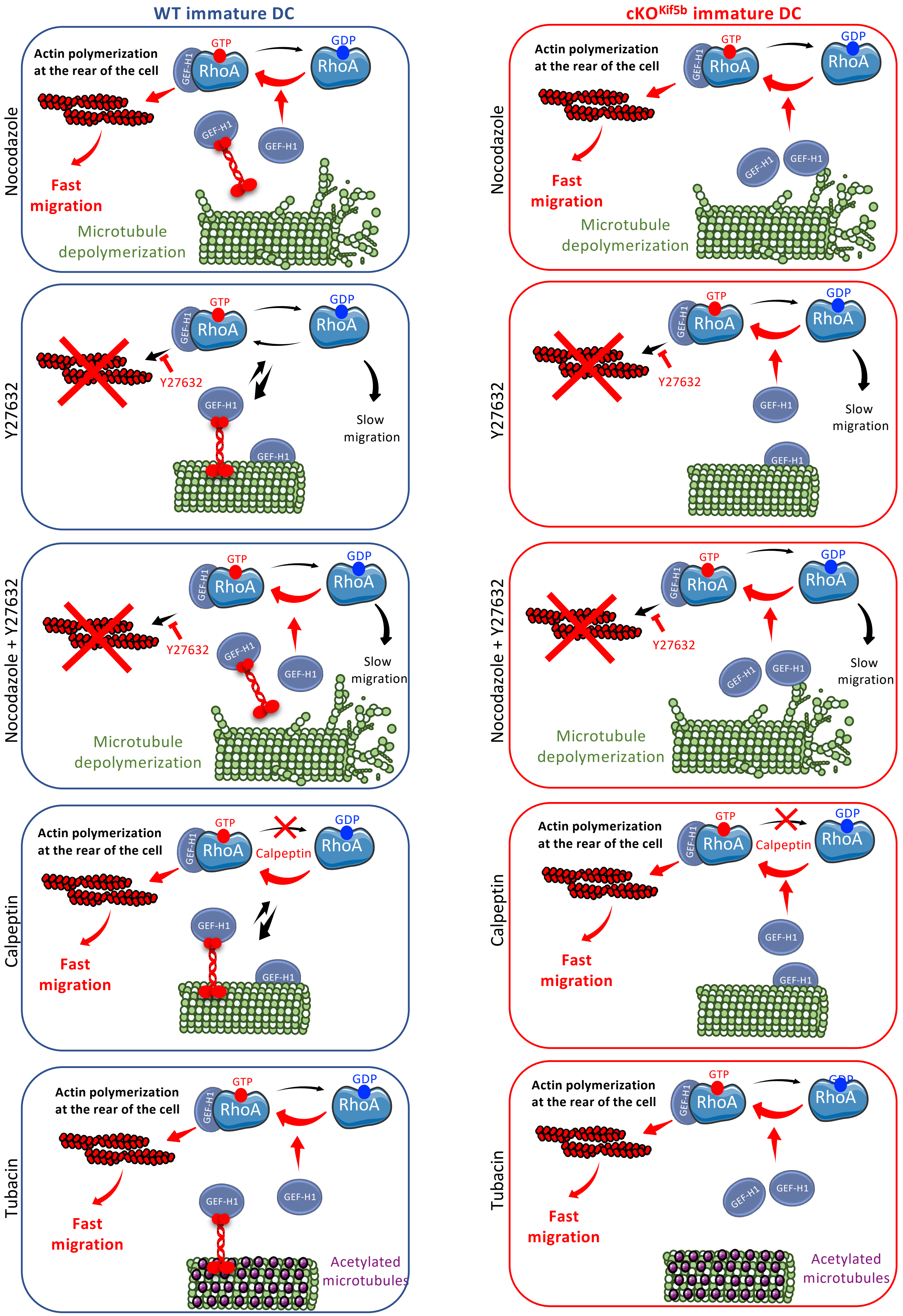

Figure S9: The suggested mechanisms of action of various drugs alone or in combination (nocodazole, Y27637, tubacin, calpeptin, and nocodazole + Y27637) on crosstalk between actin and microtubule cytoskeletons in immature WT or cKO^{Kif5b} DCs during migration in a confined environment. Created using Servier Medical

Art; licensed under CC BY 4.0 (https://creativecommons.org/licenses/by/4.0/).

Supplementary Movie 1: Migration of immature WT BMDC in 8-μm-wide microchannel

Supplementary Movie 2: Migration of immature cKO^{Kif5b} BMDC in 8-μm-wide microchannel

Supplementary Movie 3: Migration of mature WT BMDC in 8-μm-wide micro-channels

Supplementary Movie 4: Migration of mature cKO^{Kif5b} BMDC in 8-μm-wide microchannels

Supplementary Movie 5: Migration of immature WT BMDC through 1.5 μm constriction

Supplementary Movie 6: Migration of immature cKO^{Kif5b} BMDC through 1.5 μm constriction

Supplementary Movie 7: Migration of mature WT BMDC through 1.5 μm constriction

Supplementary Movie 8: Migration of mature cKO^{Kif5b} BMDC through 1.5 μm constriction

Supplementary Movie 9: Migration of immature cKO^{Kif5b} BMDC treated with Y27632 in 8-μm-wide microchannel

Supplementary Movie 10: Migration of immature cKO^{Kif5b} BMDC treated with Y27632 through 1.5 μm constriction

Supplementary Movie 11: Migration of immature WT BMDC infected with shRNA^{sc} in 8-μm-wide microchannel

Supplementary Movie 12: Migration of immature cKO^{Kif5b} BMDC infected with shRNA^{sc} in 8-μm-wide microchannel

Supplementary Movie 13: Migration of immature WT BMDC infected with shRNA$^{GEF\text{-}H1}$ in 8-μm-wide microchannel

Supplementary Movie 14: Migration of immature cKO^{Kif5b} BMDC infected with shRNA$^{GEF\text{-}H1}$ in 8-μm-wide microchannel

Supplementary Movie 15: Migration of MDDC in 8-μm-wide microchannel

Supplementary Movie 16: Migration of MDDC treated with LPS in 8-μm-wide microchannel

Supplementary Movie 17: Migration of MDDC treated with nocodazole in 8-μm-wide microchannel

Supplementary Movie 18: Migration of MDDC treated with calpeptin in 8-μm-wide microchannel

Supplementary Movie 19: Migration of MDDC treated with tubacin in 8-μm-wide microchannel

Supplementary Movie 20: Migration of MDDC infected with shRNA^{sc} in 8-μm-wide microchannel

Supplementary Movie 21: Migration of MDDC infected with shRNA^{KIF5B} in 8-μm-wide microchannel
